# Inferring Dealer Networks in the Foreign Exchange Market Using Conditional Transfer Entropy: Analysis of a Central Bank Announcement

**DOI:** 10.3390/e26090738

**Published:** 2024-08-29

**Authors:** Aleksander Janczewski, Ioannis Anagnostou, Drona Kandhai

**Affiliations:** 1 Computational Science Lab, University of Amsterdam, Science Park 904, 1098 XH Amsterdam, The Netherlands; 2 Quantitative Analytics, Financial Markets, ING Bank, Foppingadreef 7, 1102 BD Amsterdam, The Netherlands; 3 Models and Portfolio Analysis Unit, European Investment Bank, 98-100, boulevard Konrad Adenauer, L-2950 Luxembourg, Luxembourg; 4 Korteweg de Vries Institute, University of Amsterdam, Science Park 105-107, 1098 XH Amsterdam, The Netherlands

**Keywords:** information theory, conditional transfer entropy, dyadic motifs, reciprocity, foreign exchange market, financial networks, information flows

## Abstract

The foreign exchange (FX) market has evolved into a complex system where locally generated information percolates through the dealer network via high-frequency interactions. Information related to major events, such as economic announcements, spreads rapidly through this network, potentially inducing volatility, liquidity disruptions, and contagion effects across financial markets. Yet, research on the mechanics of information flows in the FX market is limited. In this paper, we introduce a novel approach employing conditional transfer entropy to construct networks of information flows. Leveraging a unique, high-resolution dataset of bid and ask prices, we investigate the impact of an announcement by the European Central Bank on the information transfer within the market. During the announcement, we identify key dealers as information sources, conduits, and sinks, and, through comparison to a baseline, uncover shifts in the network topology.

## 1. Introduction

The FX market, with daily turnovers exceeding USD 6 trillion, is a vital pillar of the global financial system. It underpins international trade by facilitating currency conversion for businesses worldwide and provides the means to mitigate currency risk by providing market participants with liquidity, thus helping to stabilize exchange rates. Furthermore, it serves as a barometer of global economic conditions, reflecting global economies’ relative strength and health. In recent years, the FX market has undergone considerable decentralization and fragmentation, and thus, it has evolved into a highly complex system. The market structure transformation has been driven by the widespread adoption of electronic trading, which enables faster and more efficient trading, but also increases the risk of flash crashes and other market disruptions. A growing number of participants, including retail investors and non-traditional actors such as hedge funds and high-frequency traders, have contributed to a more interconnected and intricate market structure. Additionally, the use of financial derivatives, such as FX futures and options, has further added to the complexity of the market.

Significant research efforts have been dedicated to unraveling complex systems and their underlying interactions in recent years [1,2,3,4,5,6,7]. Various approaches, including information-theoretic methods, have been proposed to better understand the underlying dynamics, interactions, and information flow within these intricate systems. One influential study by Bardoscia et al. [8] investigated pathways towards instability in financial networks. Their work highlighted the importance of understanding the interactions between components in complex systems and presented a framework to assess the stability of financial systems and the contagion mechanisms that can lead to cascading failures. In a different context, a study that introduced multivariate transfer entropy as a model-free measure of effective connectivity in the neurosciences was conducted by Novelli et al. [9]. The work showcases the broad applicability of information-theoretic approaches in various fields, and through the utilization of transfer entropy, they were able to identify the directed flow of information within neural networks, providing a deeper understanding of the interactions and communication within these systems. Building on the potential of information-theoretic methods, Quax, Kandhai, and Sloot [10] employed a metric called information dissipation to detect early-warning signals for financial market instability, specifically the Lehman Brothers collapse. Their findings demonstrated the potential of information-theoretic methods in identifying systemic risks, which could be crucial in preventing future financial crises and enhancing regulatory measures. In addition to the aforementioned applications, information theoretic metrics in finance have been employed for a variety of purposes, ranging from analyzing cryptocurrency returns [11] to exploring the impact of extreme external events in stock market data [12] and assessing market depth and liquidity in high-frequency trading [13].

Despite the promising potential of the information-theoretic approach, exposing the information flows in the FX market presents several challenges. First of all, the market is composed of multiple market participants with different objectives and strategies, and the data that are used to inform prices are often heterogeneous and noisy. Secondly, the interactions between market participants and between different markets can be complex, making it difficult to understand how information flows through the market and how it affects prices. Finally, the FX market is often non-linear, meaning that small changes in one part of the market can have large effects elsewhere, which makes it difficult to predict how prices will respond to changes in information.

The transmission of information in decentralized financial markets through trade has been primarily studied using model-based approaches. Wolisky [14] was among the first to explore this phenomenon, employing a simple agent-based model where trade interactions among market participants with asymmetric information could convey valuable insights to less informed agents. Following Wolinsky’s work, Duffie and Manso [15] introduced the concept of information percolation, proposing an agent-based model where market equilibrium results in full information revelation through trading. They expanded their model in subsequent publications, Duffie et al. [16,17], to include factors such as varying search intensities for new information and the influence of public information on information sharing dynamics. In [17], they further examined information percolation in segmented markets by introducing the concept of agent “connectivity”, representing the frequency of bilateral trading opportunities among agents.

To the best of our knowledge, this study represents the first investigation of FX dealer–network interactions using an information-theoretic network inference algorithm. In this paper, we contribute to the literature by providing an information-theoretic approach that offers the means to infer directional information flows between the markets. There is a wide array of information theoretic metrics available, such as mutual information, which measures the overall dependency between variables, or the recently proposed structural entropy [18], which captures the complexity and structural diversity of network communities. In this study, however, we chose to utilize transfer entropy, which was proposed by Schreiber [19], building upon the concept of Shannon entropy [20]. Transfer entropy was introduced to overcome the challenges posed by mutual information metric [21,22]: the fact that mutual information is a symmetric metric, and that it does not account for the shared history or common external driving forces [23,24]. This is particularly important when one considers Markov processes, as the previous state of the process may provide information about the future state. Transfer entropy allows for detecting and quantifying observational causal, in a probabilistic sense [19,25], nonlinear relationships between variables in complex systems, which, in the context of the FX market, can be treated as quantifying information flows between dealers [23]. As Schreiber states, the attractiveness of transfer entropy is rooted in the fact that there is no need to make any assumptions about the dynamics of the system that one wants to investigate, thus making it a truly generic metric [19].

We employ a network inference algorithm to construct networks of information flow between dealers, measured with conditional transfer entropy. To characterize the network, we analyze the temporal evolution of dealer-specific metrics, such as total inflows and outflows, and network-specific metrics, including weighted reciprocity and counts of dyadic motifs. To determine the relative position of the topological metrics observed in the hours leading up to the announcement within their distributions, we use the Z-score, also known as the standard score. The Z-score indicates the number of standard deviations an observation is from the mean. Our analysis of the network on the day of the European Central Bank’s (ECB) announcement, 12 March 2020, reveals nuanced changes in the network topology, with significant deviations from the baseline behavior in several key dealer-specific and network metrics. The results suggest increased activity of the dealers in the hours leading up to the announcement but not during the announcement itself.

The rest of the paper is organized as follows: first, we introduce the data and data processing steps along with the information-theoretic background. Next, in the results section, we present the information flow network for the baseline period of 27 February 2020 to 11 March 2020. We then perform a qualitative assessment of the changes in network topology, followed by a quantitative assessment and discussion of the results.

## 2. Materials and Methods

### 2.1. Data

This research was conducted using a high-frequency FX spot rate EUR/USD data set, with data collected at a precision of 1 millisecond. The data set comprises privately collected, irregularly spaced, temporal data of FX rates from a selection of different dealers, provided to us by courtesy of ING Netherlands for research purposes. Each data point includes two distinct time stamps: the original timestamp that is assigned at the origin by the dealer posting the quote (Timestamp1) and the timestamp set by ING when the quote update is received in the system (Timestamp2).

The EUR/USD data set comprises 109,449,980 high-frequency observations of the best bid and ask prices quoted by eight parties between 27 February 2020 and 27 March 2020. Our investigation is based on the period between 27 February 2020 and 12 March 2020, encompassing 11 trading days. The first 10 trading days, from 27 February to 11 March, are used to generate baselines, and 12 March is investigated as compared to the baseline. The period from 13 March 2020 to 27 March 2020 is used solely to perform an additional algorithm robustness and reliability analysis presented in the Appendix A.

In the investigation, we distinguish three types of dealers: the market makers (M), banks (B), and electronic trading platforms (E). Note that while banks often serve as market makers as well, we differentiate between banks and non-bank market makers in this context.

### 2.2. Data Processing

To prepare the high-frequency FX spot rate data for the analysis, we transformed it into a regularly spaced time series with a consistent sampling frequency across all subsamples propagating the last valid observation forward. This approach, also known as forward fill, is selected to ensure that no information from the future is propagated backward in time.

As our objective is to accurately reveal the information flow from dealers’ perspective, we chose to analyze the data using the timestamp assigned at the origin by the dealer who posts the quote (Timestamp1). To ensure that our results are not distorted by the electronic signal latency (the time it takes for an electronic signal to travel from its origin to destination) or ING and dealers’ clocks asynchronization, we analyzed the differences between the two timestamps assigned to each quote. By comparing the time evolution of the differences between Timestamp1 and Timestamp2, we ensured that the latencies stayed approximately constant over time in all data sets. Given that the highest latency observed was no larger than 100 milliseconds, we concluded that the sampling period of 100 milliseconds was sufficient for all dealers to observe other dealers’ updates and take action. Therefore, we resampled the data to 100 milliseconds. For a more detailed discussion on the data preprocessing methods and latency adjustment considerations, please refer to Section S1.1 in the Supplementary Materials [26].

With the aim of determining the optimal interval for daily sampling, we investigated the average intraday quote frequency for each dealer within each dataset independently. By doing, so we uncovered how the average intraday quote frequency of dealers evolves throughout the day. The highest average quote frequency was observed during the overlapping period of the London and New York sessions. Based on these observations, we opted to sample from each day eight-hour-long time windows between 8:00 and 16:00 on consecutive days, as this interval exhibits, on average, the highest trading activity in the EUR/USD data set. Further information on the intraday quote frequency is available in Section S2.3 of the Supplementary Materials [26].

Afterwards, each time series was differenced to ensure stationarity and split into five-minute-long subsamples. Effectively, an 8 h long time window yields 96 5-min-long subsamples. Consequently, the resampling process of 10 trading days yielded 960 subsamples for the EUR/USD baseline. Out of 960 subsamples, 20 were excluded due to an insufficient number of updates in the dealers’ quotes within the five-minute time window. Additionally, only in 8 subsamples were all dealers not included in the analysis due to some of them having insufficient number quote updates.

In the information-theoretic network inference algorithm, apart from transforming data into stationary time series, Kraskov et al. [27] suggest to standardize the time series to zero mean and unit variance; this approach is widely accepted by the scientific community [28,29]. After the time series is standardized, we also introduce very low-amplitude noise to the data, since for double-precision floating-point operations, Kraskov et al. [27] suggest to add noise of order $10^{-10}$. This treatment is essential when one works with empirical data with limited precision, potentially resulting in many points having equal values. Consequently, this would lead to breaking the assumption of continuously distributed points and result in spurious estimates [27]. Adding noise to the time series introduces very slight stochasticity to the Kraskov, Stögbauer, and Grassberger (KSG) estimator. However, if the information transfer is significant, it will remain significant after the addition of the noise [30].

### 2.3. Information-Theoretic Metrics

To quantify the information flows in the FX dealer network, we developed an information-theoretic network inference algorithm that utilizes two metrics: transfer entropy (also known as apparent entropy) and conditional transfer entropy. Transfer entropy, introduced by Schreiber [19], quantifies the amount of information that the past states of a source process contribute to predicting the future state of a target process, while accounting for the information about the future already ingrained in the target’s past states. In the context of Markov chains, transfer entropy can be interpreted as a measure of deviation from the generalized Markov property that can be computed with Kullback–Leibler divergence. Hence, the transfer entropy between two continuous processes from $X_{t}$ (source process) to $Y_{t}$ (target process) with time lag of 1 is defined as Kullback–Leibler divergence between two transitional probabilities; the one conditioned on the past states of both target and source processes $f(y_{t+1}|y_{t}^{\left(d_{y}\right)},x_{t}^{\left(d_{x}\right)})$ and the one that only accounts for the past states of the target process $f(y_{t+1}|y_{t}^{\left(d_{y}\right)})$. Continuous transfer entropy can be computed with the following formula:

(1) $$\begin{array}{cc} {\mathrm{TE}}_{X_{t}\to Y_{t}}^{\left(d_{y},d_{x}\right)} & ≜D\left(f\left(y_{t+1}|y_{t}^{\left(d_{y}\right)},x_{t}^{\left(d_{x}\right)}\right)\left|\right|f\left(y_{t+1}|y_{t}^{\left(d_{y}\right)}\right)\right)\\ \; & =\int_{R^{d_{x}}}\int_{R^{d_{y}}}\int_{R}f(y_{t+1},y_{t}^{\left(d_{y}\right)},x_{t}^{\left(d_{x}\right)})\log\left(\frac{f(y_{t+1}|y_{t}^{\left(d_{y}\right)},x_{t}^{\left(d_{x}\right)})}{f(y_{t+1}|y_{t}^{\left(d_{y}\right)})}\right)dy_{t+1}dy_{t}^{\left(d_{y}\right)}dx_{t}^{\left(d_{x}\right)}, \end{array}$$

where $f(y_{t+1},y_{t}^{\left(d_{y}\right)},x_{t}^{\left(d_{x}\right)})$ represents the joint probability density function, and $\int_{R^{D}}$ denotes the D-dimensional integral over the support of the variable [31]. Vector $y_{t}^{\left(d_{y}\right)}$ represents past $d_{y}$ states of a process $y_{t}$, where $d_{y}$ is the history length of the process also called the embedding dimension. By choosing some finite history length of the process, we are making an assumption that the process considered is a Markov process of order *d*; hence, it is conditionally independent of the states of history further than the past *d* states. Analogously, $d_{x}$ represents the history lengths of processes $X_{t}$. The methodology for choosing the optimal embedding dimension is further outlined in the Algorithm section.

To compute the conditional transfer entropy, we need to extend the transfer entropy formula to account for potential contributions from another causal information contributor process, say $Z_{t}$. Consequently, the formula for continuous transfer entropy is as follows:

(2) $$\begin{array}{cc} {\mathrm{CTE}}_{X_{t}\to Y_{t}|Z_{t}}^{\left(d_{y},d_{x},d_{z}\right)} & ≜D\left(f\left(y_{t+1}|y_{t}^{\left(d_{y}\right)},x_{t}^{\left(d_{x}\right)},z_{t}^{\left(d_{z}\right)}\right)\left|\right|f\left(y_{t+1}|y_{t}^{\left(d_{y}\right)},z_{t}^{\left(d_{z}\right)}\right)\right)\\ \; & =\int_{R^{d_{z}}}\int_{R^{d_{x}}}\int_{R^{d_{y}}}\int_{R}f(y_{t+1},y_{t}^{\left(d_{y}\right)},x_{t}^{\left(d_{x}\right)},z_{t}^{\left(d_{z}\right)})\log\left(\frac{f(y_{t+1}|y_{t}^{\left(d_{y}\right)},x_{t}^{\left(d_{x}\right)},z_{t}^{\left(d_{z}\right)})}{f(y_{t+1}|y_{t}^{\left(d_{y}\right)},z_{t}^{\left(d_{z}\right)})}\right)dy_{t+1}dy_{t}^{\left(d_{y}\right)}dx_{t}^{\left(d_{x}\right)}dz_{t}^{\left(d_{z}\right)}, \end{array}$$

where vector $z_{t}^{\left(d_{z}\right)}$ represents the past $d_{z}$ states of a process $Z_{t}$—the process which could potentially also reduce the uncertainty about the future state of the target process. By conditioning on process $Z_{t}$, we can filter out its contributions, and thus determine the unique information flow from process $X_{t}$ to $Y_{t}$. We note that while conditioning does eliminate redundancy, it may also introduce synergistic contributions from multivariate interactions between the conditional variables and the target variable. In our analysis, we observed a substantial reduction in the average weights of the information flow when conditional transfer entropy (CTE) is considered instead of transfer entropy (TE). This reduction suggests that conditioning effectively filtered out influences from other information contributors, leaving unique contributions from each dealer. Although it is uncertain whether any synergistic interactions were captured, the fact that all CTE edges are significantly smaller than the TE edges implies that, overall, more redundant information was filtered out than synergistic information introduced.

Both transfer entropy and conditional transfer entropy can also be represented as the conditional time-delayed mutual information in the following forms:

(3) $$\begin{array}{cc} {\mathrm{TE}}_{X_{t}\to Y_{t}}^{\left(d_{y},d_{x}\right)} & =I(Y_{t+1};X_{t}^{\left(d_{x}\right)}|Y_{t}^{\left(d_{y}\right)}), \end{array}$$

(4) $$\begin{array}{cc} {\mathrm{CTE}}_{X_{t}\to Y_{t}|Z_{t}}^{\left(d_{y},d_{x},d_{z}\right)} & =I(Y_{t+1};X_{t}^{\left(d_{x}\right)}|Y_{t}^{\left(d_{y}\right)},Z_{t}^{\left(d_{z}\right)}), \end{array}$$

where *I* denotes the mutual information.

The estimation of apparent and conditional transfer entropies requires the approximation of two transitional and one joint probability densities of the involved processes, typically based on a single realization of these processes. In our network inference algorithm, we estimate continuous apparent and conditional transfer entropies using nearest-neighbor-based Kraskov, Stögbauer, and Grassberger (KSG) algorithm I, following the methodology of Kraskov [27] and Frenzel and Pompe [32], respectively. The KSG algorithm has recently received much attention, especially in neuroscience, given that it is numerically unbiased for finite samples and relatively robust in high-dimensional settings [28,33,34]. With the developments in KSG algorithms, for example, in an extension of the algorithm to conditional mutual information by Frenzel and Pompe [32], the application of transfer entropy to network inference algorithm has recently gained much attention, especially in bioinformatics and neuroscience [35].

Transfer entropy is estimated with KSG algorithm I, which takes the following form:

(5) $$\begin{array}{cc} \hat{I}{\left(Y_{t+1};X_{t}^{\left(d_{x}\right)}|Y_{t}^{\left(d_{y}\right)}\right)}_{KSG(1)} & =\psi \left(K_{Y_{t+1}X_{t}^{\left(d_{x}\right)}Y_{t}^{\left(d_{y}\right)}}\right)+\langle \psi \left(n_{Y_{t}^{\left(d_{y}\right)}}+1\right)-\psi \left(n_{Y_{t+1}Y_{t}^{\left(d_{y}\right)}}+1\right)-\psi \left(n_{X_{t}^{\left(d_{x}\right)}Y_{t}^{\left(d_{y}\right)}}+1\right)\rangle , \end{array}$$

where $\psi \left(x\right)=\frac{\Gamma^{\prime }\left(x\right)}{\Gamma (x)}$ is the digamma function—the derivative of the log of the gamma function [27]. Furthermore, $n_{Y_{t}^{\left(d_{y}\right)}}$ represents the number of neighbors found in space $\left(Y_{t}^{\left(d_{y}\right)}\right)$, 〈⋯〉 denotes the average over all data points, and $K_{Y_{t+1}X_{t}^{\left(d_{x}\right)}Y_{t}^{\left(d_{y}\right)}}$ represents the *K*-th nearest-neighbor that is used to determine the distances in the joint space $\left(Y_{t+1},X_{t}^{\left(d_{x}\right)},Y_{t}^{\left(d_{y}\right)}\right)$.

KSG algorithm I can also be employed to estimate the conditional transfer entropy. The formula in Equation (5) can be extended to a condition on an additional source of information, say $Z_{t}$ with past $d_{z}$ states, as follows:

(6) $$\begin{array}{cc} \hat{I}{\left(Y_{t+1};X_{t}^{\left(d_{x}\right)}|Y_{t}^{\left(d_{y}\right)},Z_{t}^{\left(d_{z}\right)}\right)}_{KSG(1)} & =\psi \left(K_{Y_{t+1}X_{t}^{\left(d_{x}\right)}Y_{t}^{\left(d_{y}\right)}Z_{t}^{\left(d_{z}\right)}}\right)\\ \; & +\langle \psi \left(n_{Y_{t}^{\left(d_{y}\right)}Z_{t}^{\left(d_{z}\right)}}+1\right)-\psi \left(n_{Y_{t+1}Y_{t}^{\left(d_{y}\right)}Z_{t}^{\left(d_{z}\right)}}+1\right)-\psi \left(n_{X_{t}^{\left(d_{x}\right)}Y_{t}^{\left(d_{y}\right)}Z_{t}^{\left(d_{z}\right)}}+1\right)\rangle . \end{array}$$

### 2.4. Algorithm

The information-theoretic network inference algorithm involves many steps outlined below.

**Step 1 Data preprocessing:** Initially, the data in a subsample are preprocessed as detailed in the Data Preprocessing section.

**Step 2 Optimal dimension and delay embedding:** Afterward, for each dealer (source) and dealer (target) pair in the subsample, we jointly optimize both the embedding dimension and delay parameters using the Ragwitz criterion [36]. The Ragwitz criterion relies on a locally constant predictor of the future state ($w_{t+1}$) of embedding vector $w_{t}^{k}$, with the future state prediction estimated from the nearest neighbors of the variable after embedding is applied [37]. For a given neighborhood diameter ϵ, the neighborhood of $w_{t}^{k}$, denoted by $U_{n}$, is defined as $U_{n}=\{w_{n}^{k}:||w_{n}^{k}-w_{t}^{k}\left||\leq \epsilon \right\}$. Using this neighborhood, the Ragwitz criterion computes a locally constant estimate ${\hat{w}}_{t+1}$ of the future state $w_{t+1}$ as follows:

(7) $$\begin{array}{c} {\hat{w}}_{t+1}=\frac{1}{|U_{n}|}\sum_{w_{n}^{k}\in U_{n}}y_{n+1}. \end{array}$$

This estimation simply amounts to taking the mean value of the future states ($w_{n+1}$) of the nearest neighbors of the embedding vector $w_{t}^{k}$. In practice, ϵ is replaced by a specific number of *K* nearest neighbors taken into account for the estimation, which serves as a “natural” substitute when the KSG algorithm is employed [28]. Next, the squared error of the local predictor is computed for each time index in the embedding vector, and the mean squared error is determined. The mean squared error is computed for each combination of embedding dimension and embedding delay parameters that one chooses to investigate. Based on Ragwitz’s criterion, the parameters that yield the smallest mean squared error are optimal. In our investigation, the maximal embedding dimension and delays were set to 4, which is simply because higher embedding dimensions would be computationally unfeasible given the number of subsamples considered.

**Step 3 Optimal source-target delay:** In the following step, for each dealer (source) and dealer (target) pair in the subsample, we determine the true delay between source and target processes following the method proposed by Wibral et al. [38]. The optimal source-target delay is determined to be the delay for which the largest transfer entropy is determined, namely:

(8) $$\begin{array}{cc} \delta & =\underset{u\in U}{\mathrm{argmax}}\left(I(Y_{t+1};X_{t-u}^{\left(d_{x}\right)}|Y_{t}^{\left(d_{y}\right)})\right), \end{array}$$

where *u* represents source–target delay, U is the investigated delay space, i.e., $U=\{0,1,2,\ldots ,u_{\max}\}$, and δ represents the true delay between source and target processes. Our algorithm examines source-target delays up to a maximum of 4, which, considering the resampling interval, is equivalent to a 400 ms delay. From our latency investigation and the literature, it is apparent that 400 ms is enough time for dealers to react to quote updates of other dealers. Furthermore, the analysis of the distribution of true source-target delays revealed that most of the true delays recovered were equal to 1.

**Step 4 Estimating transfer entropy:** In the fourth step of our approach, the transfer entropy from the source dealer to the target dealer is estimated using KSG algorithm I (Equation (5)). First, we concatenate the vectors of all processes $\left(Y_{t+1},X_{t}^{\left(d_{x}\right)},Y_{t}^{\left(d_{y}\right)}\right)$ to create a joint embedding space. Next, we determine the Chebyshev distance to the *K*-th nearest neighbor (in our case, the 8th nearest neighbor) for each time point using a modified Scipy’s KD-tree algorithm [39]. Then, we use the obtained distance to identify the number of nearest neighbors, i.e., $n_{Y_{t}^{\left(d_{y}\right)}}$, $n_{Y_{t+1}Y_{t}^{\left(d_{y}\right)}}$, and $n_{X_{t}^{\left(d_{x}\right)}Y_{t}^{\left(d_{y}\right)}}$ in the respective spaces $\left(Y_{t}^{\left(d_{y}\right)}\right)$, $\left(Y_{t+1},Y_{t}^{\left(d_{y}\right)}\right)$, $\left(X_{t}^{\left(d_{x}\right)}Y_{t}^{\left(d_{y}\right)}\right)$. This process is repeated for all vectors in the subsample to calculate the average number of nearest neighbors found for each distance in each space, as in Equation (5). This yields one transfer entropy estimate for a single pair of (source) dealer and (target) dealer.

*K* effectively controls the bias of the estimation, which here scales with a factor K/N [27]. Based on the results from the literature and numerical experiments with the FX data, we choose K=8, which very significantly reduced the variance of the estimate while maintaining negligible systematic errors. This choice was also motivated by the large variance of transfer entropy estimates with K=4.

**Step 5 Statistical testing:** Afterward, we perform permutation testing to determine the statistical significance of the transfer entropy estimates. When performing the statistical assessment with the permutation technique, we are essentially assessing whether the estimated Kullback–Leibler divergence between transitional probabilities $f(y_{t+1}|y_{t}^{\left(d_{y}\right)})$ and $f(y_{t+1}|y_{t}^{\left(d_{y}\right)},x_{t}^{\left(d_{x}\right)})$ is indeed statistically significant. We test for the null hypothesis that the state changes $y_{t}^{\left(d_{y}\right)}\to y_{t+1}$ have no temporal dependence on the source process $x_{t}^{\left(d_{x}\right)}$ [40], hence:

(9) $$\begin{array}{c} H_{0}:f\left(y_{t+1}|y_{t}^{\left(d_{y}\right)}\right)=f\left(y_{t+1}|y_{t}^{\left(d_{y}\right)},x_{t}^{\left(d_{x}\right)}\right)\Rightarrow {\hat{\mathrm{TE}}}_{X_{t}\to Y_{t}}^{\left(d_{y},d_{x}\right)}=0, \end{array}$$

(10) $$\begin{array}{c} H_{1}:f\left(y_{t+1}|y_{t}^{\left(d_{y}\right)}\right)\neq f\left(y_{t+1}|y_{t}^{\left(d_{y}\right)},x_{t}^{\left(d_{x}\right)}\right)\Rightarrow {\hat{\mathrm{TE}}}_{X_{t}\to Y_{t}}^{\left(d_{y},d_{x}\right)}>0. \end{array}$$

The one-sided alternative hypothesis is motivated by the fact that transfer entropy is a non-negative measure. To test the above-presented hypotheses, we generate an empirical distribution of transfer entropy estimates under a null hypothesis. We achieve this by generating a large number of source process surrogates $\left(X_{t}^{s}\right)$, which preserve transitional probability $p(y_{t+1}|y_{t}^{\left(d_{y}\right)})$, but destroy the dependence in $p(y_{t+1}|y_{t}^{\left(d_{y}\right)},x_{t}^{\left(d_{x}\right)})$ [28]. We generate the *S* of source process surrogates $\left(X_{t}^{s}\right)$ by shuffling the vectors of past states $x_{t}^{\left(d_{x}\right)}$ among the set of $\left\{y_{t+1},y_{t}^{\left(d_{y}\right)},x_{t}^{\left(d_{x}\right)}\right\}$ tuples, i.e., interchanging their time indices within the time-series, as proposed by Lizier [28]. Then, the *p*-value is determined by simply counting the number of cases when ${\hat{\mathrm{TE}}}_{X_{t}^{s}\to Y_{t}}^{\left(d_{y},d_{x}\right)}>{\hat{\mathrm{TE}}}_{X_{t}\to Y_{t}}^{\left(d_{y},d_{x}\right)}$, i.e.,

(11) $$\begin{array}{c} p-\mathrm{value}=\frac{1}{S}\sum_{i=1}^{S}1\left({\hat{\underset{i}{\mathrm{TE}}}}_{X_{t}^{s}\to Y_{t}}^{\left(d_{y},d_{x}\right)}>{\hat{\mathrm{TE}}}_{X_{t}\to Y_{t}}^{\left(d_{y},d_{x}\right)}\right), \end{array}$$

where 1(·) denotes the indicator function, and *S* is the number of surrogate source processes. For a given significance level α, we reject $H_{0}$ if the *p*-value <α [40]. Additionally, one should note that since we are going to conduct multiple hypothesis tests, we need to employ Bonferroni correction. In our research, *S* was set to 500 because this is the largest number of permutations that was feasible given our computational limitations. Note that we computed a total of 107,520 entropy estimates. Thus, with 500 permutations for each estimate, we are looking at computing approximately up to 54 million entropy estimations, which is quite a large number. Steps 2–5 are repeated for all possible source and target dealer pairs in a given subsample.

**Step 6 Estimating conditional transfer entropy:** For all statistically significant transfer entropy pairs between source and target dealers, we recompute the transfer entropy while conditioning on all other statistically significant information contributors to the source process. To accomplish this, we essentially repeat Steps 2–5 for statically significant information flows, with the key difference being that we now also account for all statistically significant information contributors identified through the initial transfer entropy calculation. Therefore, in this step, KSG algorithm I for the conditional transfer entropy estimation is employed (Equation (6)). This approach allows us to filter out redundant information flows and focus on the unique information that flows from source and target dealers. For more details on the network inference algorithm, please refer to Sections S1.2 and S1.3 in the Supplementary Materials [26].

### 2.5. Weighted Reciprocity

The weighted reciprocity of the network is a metric that quantifies the extent to which bilateral connections in a weighted directed network are reciprocated. Let us denote the weight of a connection from node *i* to node *j* as $w_{ij}$. Then, the reciprocity between the two nodes is a measure of how much the interaction from *j* to *i* ($w_{ji}$) mirrors the interaction from *i* to *j* ($w_{ij}$), i.e., $\min(w_{ij},w_{ji})$ [41]. The weighted reciprocity of the network can be computed with the following formula [41]:

(12) $$\begin{array}{c} r_{w}=\frac{\sum_{i}\sum_{i\neq j}\min\left(w_{ij},w_{ji}\right)}{\sum_{i}\sum_{i\neq j}w_{ij}}. \end{array}$$

The weighted reciprocity of the network can range between 0 and 1, where 0 indicates no reciprocated connections and 1 represents perfect reciprocity of all edges.

### 2.6. Validation and Code Availability

We validated the transfer entropy metric with the analytical solution provided by Kaiser and Schreiber [42]. Additional details regarding the validation process and its results can be found in Section S3 of the Supplementary Materials [26].

The code associated with this research, including the information-theoretic network inference algorithm, is developed in C++ Version 2.1 and is open-source. It can be accessed through the following link: Github. For detailed information on the network inference algorithm and its implementation, we refer the reader to Sections S1.2 and S1.3 of the Supplementary Materials [26].

## 3. Results

In this section, we first establish a baseline by introducing the network information flow map for the period from 27 February to 11 March 2020. Next, we perform a qualitative assessment of the changes in the network’s topology on 12 March 2020, the day of the ECB announcement. This is followed by a quantitative evaluation of the deviations in the dealers’ total inflows, outflows, and the weighted reciprocity of the dealer network using Z-scores. Finally, we shift our focus to the binary representation of the network, analyzing the counts of dyadic motifs, including bilateral, unilateral, and missing connections.

### 3.1. Baseline—27 February to 11 March

**Figure 1:**
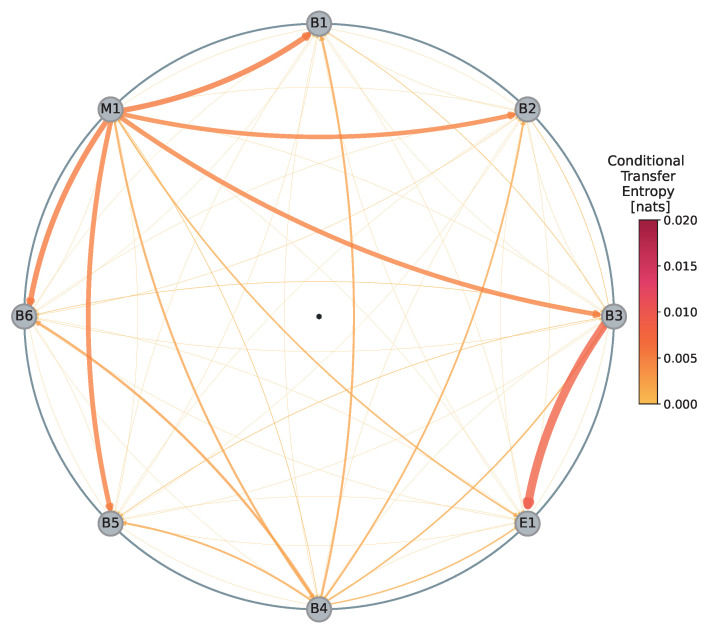
Information flow map illustrating the mean information flows in the EUR/USD dealer network for the period from 27 February to 11 March 2020. Each dealer is represented as a node, and their positions on the circle are arbitrary. The thickness and color of the directional edges between dealer pairs are proportional to the conditional transfer entropy and are expressed in nats.

In this section, we present the results of our investigation into the flow of information within the EUR/USD dealer network during the period from 27 February to 11 March 2020. Figure 1 illustrates the mean representation of the network and information flows within this period of time. Specifically, the flows (directional edges) depicted represent the average of family-wise, statistically significant (at the 10% level) conditional transfer entropies (CTEs) computed for each pair of dealers during the specified period.

In the baseline network (Figure 1), we observe numerous directional connections between dealers, with varying degrees of strength. Notably, dealer M1 exhibits the highest total information outflows, clearly highlighted by many pronounced edges directed away from it. The total outflow from dealer M1 is determined to be 307.4 nats (note that all values of CTE presented in this article represent $10^{-4}$ nats as indicated in Table 1). At the same time, dealer M1 appears to receive the least amount of information on average, with total inflows of only 19.5 nats. This observation suggests that M1 is the dominant source of unique information in the network, primarily contributing to dissemination of unique information. Another interesting behavior is observed for dealer B3, which appears to play a central role as a conduit for information to dealer E1. B3 is characterized by total inflows of 81.0 nats and outflows of 123.3 nats, indicating that while B3 receives a substantial amount of information, it also transmits even more of it to other dealers. Turning our attention to dealer E1, we see it primarily absorbs information, as shown by its total inflows of 127.4 nats (with 93.9 nats coming from B3) and minimal outflows of only 8.4 nats. This implies that E1 predominantly acts as an information sink, absorbing information without significantly contributing to its propagation within the network. Thus, based on the above observations, we can clearly distinguish the roles of different dealers in the network: those that dominate in terms of unique information transmission, those that act as intermediaries, and those that predominantly absorb information, the followers.

### 3.2. Qualitative Assessment of Changes in the Network’s Topology on 12 March 2020

In January 2015, the Governing Council of the ECB announced an expanded Asset Purchase programme (APP) as part of its monetary policy measures to stimulate economic activity and increase inflation in the Eurozone. The program started in March 2015 and since then has been adjusted several times. (More information about the history and evolution of the ECB’s Asset Purchase Programme can be found on ECB’s website [43]). The APP can be considered a form of Quantitative Easing, a monetary policy strategy commonly employed by central banks globally [44,45]. Announcements of asset purchases may have an impact on exchange rates, with evidence pointing to announcement and expectation effects that tend to depreciate the Euro [46,47,48]. In this paper, we focus on obtaining an understanding of how information on asset purchases is getting processed by the market rather than studying the long term effects on the level of the exchange rates.

On 12 March 2020 at 13:30 GMT time, the ECB announced an effective expansion of the APP in the form of a temporary envelope of additional net asset purchases of EUR 120 billion [49,50]. The aim of this measure was to promote favorable financing conditions for the actual economy during a period of elevated uncertainty, as the COVID-19 pandemic was spreading rapidly around the world and causing widespread economic disruption. In the rest of this section, we present the results of our analysis of the information flows in the EUR/USD dealer network during the period surrounding the ECB’s announcement.

Previous research conducted by Hagströmer and Menkveld [51] shows that public announcements can reduce the incentive for dealers to seek information. Consequently, we postulate that the ECB announcement would result in a reduction in information flows in the dealer network.

**Figure 2:**
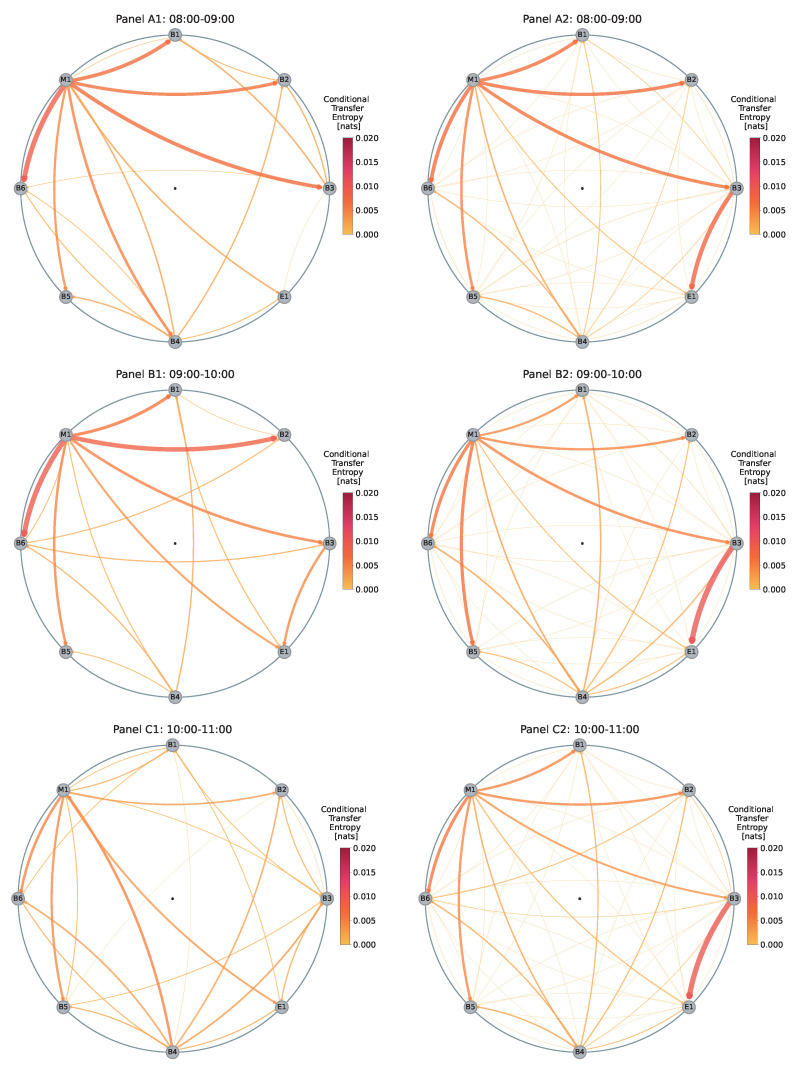

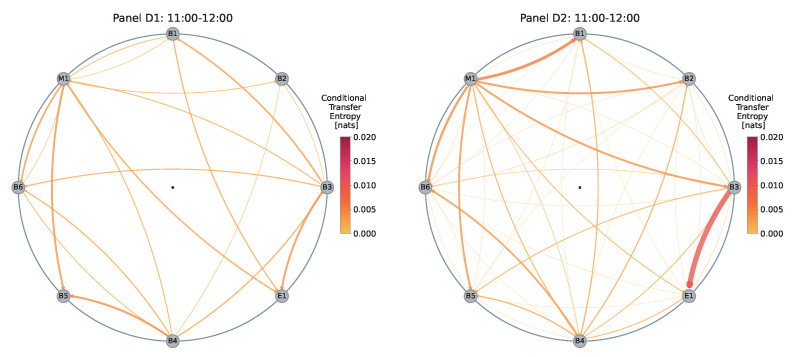
Information maps illustrating changes in the EUR/USD dealer network between 8:00 and 12:00 on 12 March 2020, the day of the ECB announcement. The panels on the left-hand side represent the information flow for each one-hour interval on the day of the announcement. Whereas, the maps on the right-hand side represent the average information flow for the same one-hour intervals, computed from the preceding 10 trading days (27 February–11 March 2020).

**Figure 3:**
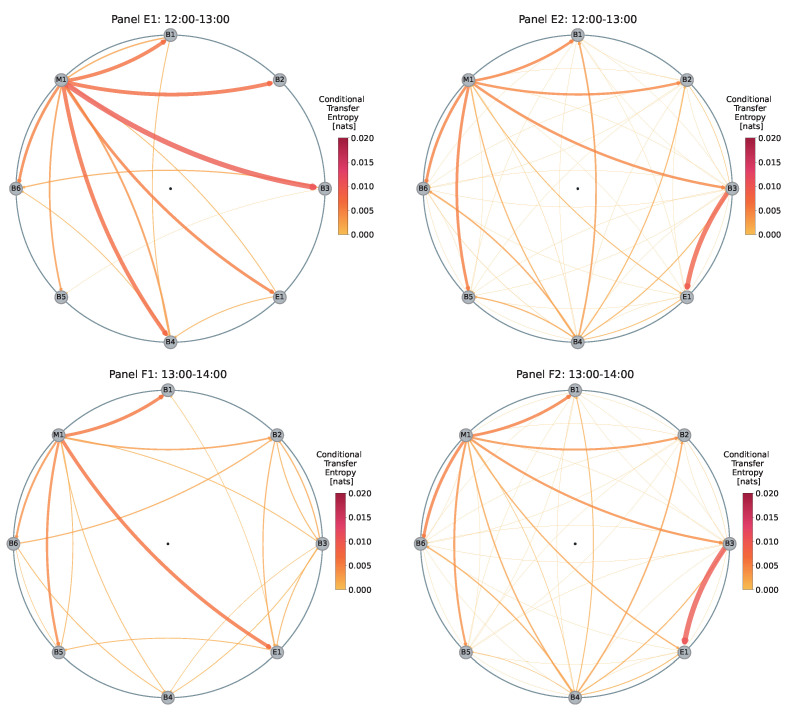

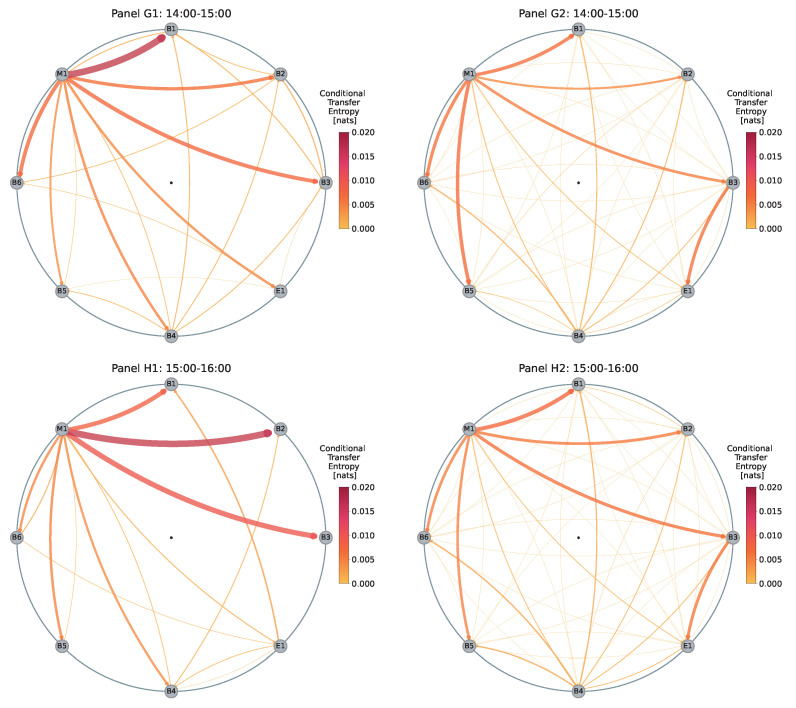
Information maps illustrating changes in the EUR/USD dealer network between 12:00 and 16:00 on 12 March 2020, the day of the ECB announcement. The panels on the left-hand side represent the information flow for each one-hour interval on the day of the announcement. Whereas, the maps on the right-hand side represent the average information flow for the same one-hour intervals, computed from 10 trading days (27 February–11 March 2020).

To examine this phenomenon, we analyze the information maps generated for each hour between 8:00 and 16:00 GMT on the day of the announcement, presented on the left-hand side of Figure 2 and Figure 3. On the right-hand side of the same figures, we juxtapose these networks with baseline networks created by averaging the same time windows over the previous 10 trading days (27 February–11 March 2020). Thus, each left-hand panel (e.g., Panel A1) represents the network for a one-hour time window on 12 March, while the corresponding right-hand panel (e.g., Panel A2) shows the average network for that same one-hour time window over the preceding 10 days.

Each average network is generated through a two-step process. First, for each trading day, we average the 12 five-minute subsamples within each one-hour period. For example, we average the 12 five-minute intervals between 9:00 and 10:00 to obtain a single hourly average for that day. Second, we take these hourly averages from the preceding 10 trading days and average them together to create a baseline representation for that specific one-hour time window.

This comparative visualization helps us understand the changing dynamics in the dealer network during the ECB announcement by directly comparing them to the average behavior at the same times on other days. By taking this approach, we aim to filter out intra-day dynamics that may obscure the significance of the deviations observed on the day of the ECB announcement.

In Panels A1 and A2 of Figure 2, we observe that between 8:00 and 9:00 h, the information map generated for the day of the event (Panel A1) has many characteristics similar to the average (baseline) network (Panel A2), with the primary difference being a smaller overall number of information flows between dealers. Moreover, dealer M1 exhibits strong information outflows to dealers B1, B2, B3, and B6, similar to the baseline for that one-hour time window. However, the strong flow from M1 to B4 appears to be atypical for this period. Additionally, dealer B3 does not exhibit a strong outflow to E1 as expected.

Panel B1 closely resembles Panel A1, except for small variations in the strengths of dealer M1’s outflows and a few minor changes in inflows and outflows among other dealers. In the baseline (Panel B2), M1’s outflows are slightly weaker than in Panel A2.

Next, Panel C1 reveals a significant decrease in the outflows from dealer M1, particularly to B1 and B2, which are much lower than typically observed. Additionally, we see a strong outflow from B4 to M1.

In Panel D1, we observe substantially lower information flows than in the previous time window (Panel C1). However, the baseline networks from the corresponding time windows (Panels C2 and D2) are nearly identical, suggesting that the observed decrease in information flows from C1 to D1 is unexpected.

Conversely, in Panel E1 of Figure 3, approximately 30 min before the ECB’s announcement, we observe the emergence of several strong information flows originating from M1, which are substantially stronger compared to the baseline in Panel E2. Additionally, the overall number of flows in the network decreases, with no outflows from dealer B2 and the absence of the information conduit from B3 to E1.

In Panel F1, which captures the changes before and during the ECB’s announcement between 13:00 and 14:00, we observe substantially fewer outflows from M1 compared to Panel E1. Only the outflows towards B1 and E1 remain relatively strong. The flow from M1 to B3 is absent, and the flow from B3 to E1 is replaced by a strong flow from M1 to E1.

Panel G1 reveals a sudden, very strong flow from M1 to B1, which is much stronger than usually observed. Other characteristics remain similar to the baseline in Panel G2, except for a substantially stronger flow from M1 to E1, a limited flow from B3 to E1, and a much stronger flow from M1 to B4.

Panel H1 highlights pronounced flows from M1 to dealers B2 and B3, which are substantially stronger than in the baseline shown in Panel H2. Additionally, B2 does not exhibit any outflows and has only two inflows. B3 has no outflows and does not serve as a conduit of information for E1, contrary to the baseline behavior.

### 3.3. Quantitative Assessment of Changes in the Network’s Topology on 12 March 2020

To quantify the differences that we qualitatively identified in the previous subsection, such as changes in the inflows and outflows of various dealers, as well as the proportion of reciprocated flows, we compute Z-scores by comparing each metric on 12 March within specific one-hour time windows to their average values within the same time windows over the past 10 trading days.

For this purpose, we calculate the mean and sample standard deviation of each metric over the 10 subsamples corresponding to one-hour intervals from the preceding 10 trading days. Specifically, for each time interval (e.g., 9:00–10:00), we use the 10 samples from the past 10 days to compute the mean and the sample standard deviation. This approach allows us to assess the significance of the deviations in dealers’ inflows and outflows, as well as the weighted reciprocity of the entire network, from the expected values of these measures.

**Figure 4:**
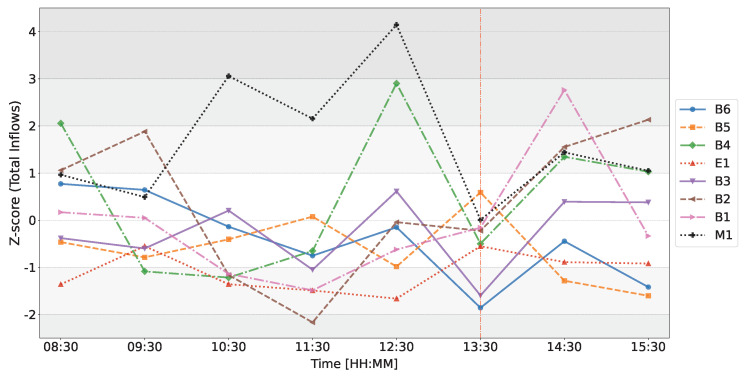
Temporal evolution of the Z-score of total inflows for each dealer on 12 March 2020 as compared to the baseline. The red line represents the ECB announcement at 13:30 GMT. Each data point is positioned at the middle of the 1-h time window in which it was generated; for example, a point at 8:30 indicates that the metric was generated for the time window from 8:00 to 9:00.

Figure 4 illustrates the changes in Z-scores of the inflows (in-strengths) of all dealers over time on the day of the ECB announcement on 12 March 2020. Focusing on the temporal evolution of M1’s Z-score of its total inflows, we observe that at 10:30 (time window from 10:00 to 11:00) the Z-score abruptly changes from Z=0.49 to Z=3.06, indicating that on the day of the announcement, M1 exhibits much higher inflows between 10:00 and 11:00 than it typically does. As previously noted, Panel C1 from Figure 2 reveals a strong abnormal flow from B4 to M1, which likely contributes to this deviation in the Z-score. M1’s Z-score of total inflows reaches its highest values in the time window of 12:00–13:00, peaking at Z=4.15, indicating significant deviations from their baseline behavior. During this period, M1 exhibits an unusually strong flow to B4, which is corroborated by B4’s abnormal total inflows with Z=2.90. As the announcement unfolds, the total inflows of all dealers appear to be back in the range of Z=−1 to Z=1, except for dealers B3 and B6. This suggests that, overall, total inflows are more reminiscent of a typical day for almost all dealers. This observation somewhat contradicts our expectations, as previous research [16,51] has shown that public announcements reduce the incentive for dealers to seek information. However, for most dealers, we do not observe a significant reduction, except for B3 and B6.

**Figure 5:**
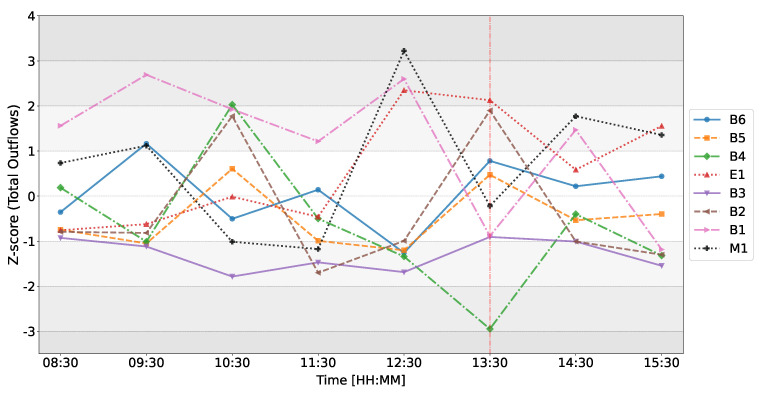
Temporal evolution of the Z-score of total outflows for each dealer on 12 March 2020 as compared to the baseline. The red line represents the ECB announcement at 13:30 GMT.

Figure 5 illustrates the changes in *Z*-scores of the total outflows (out-strengths) of all dealers over time on the day of the ECB announcement on 12 March 2020. In this figure, we observe significant deviations for various dealers. For instance, in the time window from 10:00 to 11:00, dealers B1, B4, and B2 exhibit substantial deviations in total outflows from the expected behaviors, with *Z*-scores of Z=1.93, Z=2.03, and Z=1.78, respectively. In the time window from 12:00 to 13:00, dealer M1 again shows the highest deviation with a *Z*-score of Z=3.22, indicating higher-than-usual total outflows. This is clearly visualized in Panel E1 of Figure 3, where we previously note much more pronounced outflows from M1. Additionally, dealers E1 and B1 also exhibit elevated total outflows preceding the announcement, with *Z*-scores of Z=2.35 and Z=2.60, respectively.

In contrast to Figure 4, where we observe that total inflows for all dealers appear rather typical in the time window from 12:00 to 13:00, Figure 5 shows that the total outflows are actually atypical for many dealers in the same time window. Specifically, E1 has a *Z*-score of Z=2.35, B2 has Z=1.90, and B4 has Z=−2.95.

The juxtaposition of *Z*-scores for total inflows and total outflows suggests that while the total inflows to dealers remained generally within the expected range, the sources of the information changed, as indicated by the unusual total outflows for some dealers. This suggests a sudden reorganization of the flows in the network preceding the ECB announcement.

**Figure 6:**
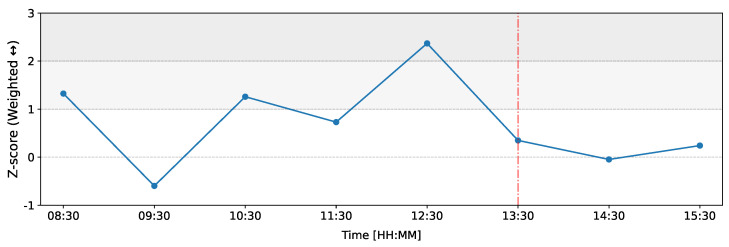
Temporal evolution of the Z-score of weighted reciprocity of the network on 12 March 2020 as compared to the baseline. The red line represents the ECB announcement at 13:30 GMT.

Next, we explore the temporal evolution of the weighted reciprocity of the network, illustrated in Figure 6. In this figure, similar to Figure 4 and Figure 5, we observe that the most abrupt deviation from the expected behavior occurs in the time window from 12:00 to 13:00, with weighted reciprocity having a *Z*-score of Z=2.37. This indicates that more flows between dealers are reciprocated during this period, suggesting that the percolation of information in the network is more bilateral rather than unilateral.

### 3.4. Quantitative Assessment of Changes in the Binary Network’s Topology on 12 March 2020

In this subsection, we shift our focus from the weighted form of the network to its binary representation. In this binary network, each connection is either present (1) or absent (0). We explore three specific dyadic motifs in this binary form: bilateral (reciprocated) connections, unilateral (nonreciprocated) connections, and no connection motifs. The following figures illustrate the changes in *Z*-scores of the counts of these motifs over time on the day of the ECB announcement on 12 March 2020.

**Figure 7:**
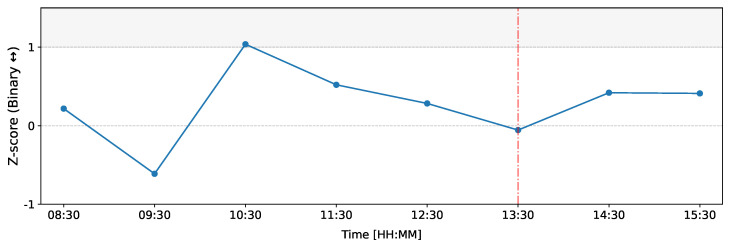
Temporal evolution of the Z-score of the count of bilateral (reciprocated) connections in the binary representation of the network on 12 March 2020 as compared to the baseline. The red line represents the ECB announcement at 13:30 GMT. Each data point is positioned at the middle of the 1 h time window in which it was generated.

Figure 7 illustrates the changes in *Z*-scores of the count of reciprocated connections (bilateral connections) over time on the day of the ECB announcement on 12 March 2020. In this figure, we observe that the most significant deviations occur at 10:30 (time window from 10:00 to 11:00) with a *Z*-score of Z=1.04, indicating a higher-than-normal number of reciprocated connections. Notably, during the same time window, dealers B1, B4, and B2 exhibit substantial deviations in total outflows (Figure 5), and M1’s total inflows show a significant increase (Figure 4). Thus, these changes in weighted reciprocity align with the observations made about total inflows and total outflows.

**Figure 8:**
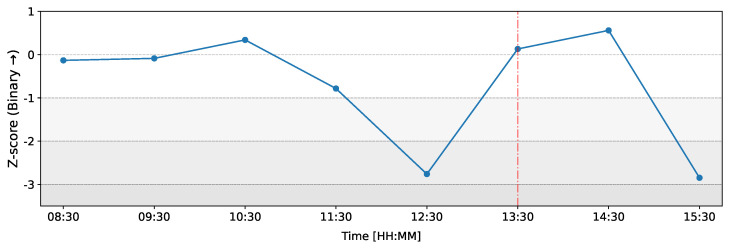
Temporal evolution of the Z-score of the count of unilateral (nonreciprocated) connections in the binary representation of the network on 12 March 2020 as compared to the baseline. The red line represents the ECB announcement at 13:30 GMT.

Figure 8 illustrates the changes in *Z*-scores of the count of nonreciprocated connections (unilateral connections) over time on the day of the ECB announcement on 12 March 2020. In this figure, the most significant deviation occurs at 12:30 (time window from 12:00 to 13:00) with a *Z*-score of Z=−2.76, indicating a much-lower-than-expected number of nonreciprocated connections. Notably, during the same time window, dealer M1 also shows the highest deviation in total outflows (Figure 5), and M1’s *Z*-score of total inflows reaches its peak value (Figure 4).

**Figure 9:**
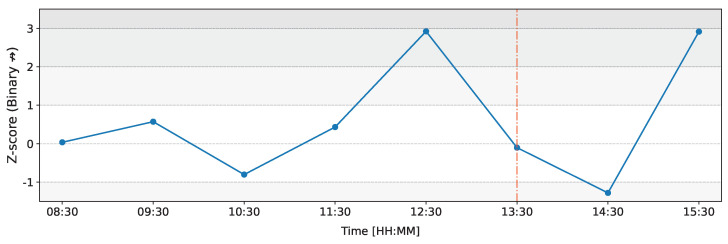
Temporal evolution of the Z-score of the count of missing connections in the binary representation of the network on 12 March 2020 as compared to the baseline. The red line represents the ECB announcement at 13:30 GMT. Each data point is positioned at the middle of the 1 h time window in which it was generated.

Figure 9 illustrates the changes in *Z*-scores of the count of missing connections over time on the day of the ECB announcement on 12 March 2020. In this figure, the most significant deviation occurs at 12:30 (time window from 12:00 to 13:00) with a *Z*-score of Z=2.92, indicating a much higher than expected number of missing connections. As previously mentioned, during the same time window, significant deviations were observed in both total inflows and outflows (Figure 4 and Figure 5).

## 4. Discussion

The analysis of the network on the day of the ECB announcement, 12 March 2020, reveals significant deviations from the baseline behavior in several key dealer-specific and network metrics. Notably, the time windows from 10:00 to 11:00 and from 12:00 to 13:00 show pronounced changes in the network’s inflows, outflows, and reciprocity.

In the 10:00 to 11:00 time window, we observe a sharp increase in M1’s total inflows with a Z=3.06 (Figure 4) and significant deviations in total outflows for dealers B1, B4, and B2 (Figure 5). Additionally, the Z-score of the count of bilateral connections in binary representation of the network reaches Z=1.04 (Figure 7), indicating a higher-than-expected number of reciprocated connections during this period. Similarly, Figure 6, which represents the temporal evolution of the Z-score of the weighted reciprocity of the network, also reveals a heightened Z-score, aligning with this observation.

The 12:00 to 13:00 time window shows the highest deviations across multiple metrics, suggesting unusual network topology and activity prior to the ECB announcement. M1’s total inflows reach a Z-score of Z=4.15, while its total outflows show a Z-score of Z=3.22 (Figure 4 and Figure 5). In Figure 4 and Figure 5, we also note that total inflows for all dealers appear rather typical in this time window, whereas the total outflows are actually unusual for many dealers. Furthermore, Figure 6 shows that in this time window, the Z-score of the weighted reciprocity of the network has Z=2.37. During this period, the Z-score for the count of the nonreciprocated connections drops significantly to Z=−2.76, and the Z-score for the count of missing connections rises sharply to Z=2.92 (Figure 8 and Figure 9). These observations suggest a reorganization of the network’s connectivity, with fewer unilateral connections and more empty connections than typically observed.

These observations suggest that most metrics indicate significant deviations at 12:00–13:00 (except for binary reciprocated connections), pointing to nuanced changes in the network topology right before the ECB announcement. Overall, the data indicate that the network’s dynamics were significantly disrupted on the day of the ECB announcement, particularly in the hours leading up to it. The heightened activity and reorganization of connections suggest an anticipation of the announcement’s impact, as dealers adjusted their positions and information flows in response to market expectations. As previous research [16,51] has shown, public announcements typically reduce the incentive for dealers to seek information; however, we do not observe such a reduction in this case, as, overall, the total inflows of all dealers remained within the norms. Therefore, our findings do not support our hypothesis that the ECB announcement would result in a reduction in information flows in the dealer network.

This analysis underscores the importance of understanding network dynamics in financial markets, particularly during significant events that can lead to abrupt changes in behavior. FX market regulators can leverage insights into the behavior of FX dealers during high-volatility events. By analyzing the flow of information and interactions among dealers during announcements or other events, regulators can better understand the risks associated with different market participants, detect potential market misconduct, and inform policy-making decisions aimed at enhancing market transparency and stability. Such insights can ultimately help regulators ensure fair and efficient markets.

## 5. Conclusions

This research is the first to analyze such an extensive, high-frequency data set, performing over a hundred thousand transfer entropy estimations. Additionally, we present the first openly available implementation of this parallelized information-theoretic network inference algorithm in a low-level language. By applying this algorithm to the EUR/USD FX dealer-network interactions related to the European Central Bank (ECB)’s asset purchase program in March 2020, we gained valuable insights into market information processing during this event. We investigated the temporal evolution of key topological metrics, including total inflows, total outflows, weighted reciprocity, and counts of dyadic motifs, providing a comprehensive analysis of the network’s dynamics on the day of the ECB’s announcement.

Building on the theories of information transmission by Wolinsky [14] and information percolation by Duffie and Manso [15], our study uncovered and quantified flows of unique information between market participants with asymmetric information and visualized the process of information transmission in a decentralized market. Additionally, we contribute to the existing literature by empirically testing Duffie et al.’s [16] theoretical prediction that information percolation abates during times of significant public information. Our results do not fully corroborate the theoretical prediction, since they suggest increased activity of the dealers in the hours leading up to the announcement but no significantly reduced activity during the announcement itself.

Finally, our findings shed light on how FX dealers process information and interact with each other, which can have significant implications for market efficiency and stability. Importantly, while our study focuses on the FX market, the proposed network inference algorithm can be applied to a wide range of complex systems where time series data are available, serving as a powerful tool for uncovering interdependencies.

## Figures and Tables

**Table 1:** Summary of average information inflows and outflows for each dealer in the EUR/USD information flow map presented in Figure 1.

	**Inflows [**$10^{-4}$ **Nats]**									
**Outflows [**$10^{-4}$ **Nats]**		**B6**	**B5**	**B4**	**E1**	**B3**	**B2**	**B1**	**M1**	**Sum**
	**B6**	-	3.1	2.9	2.0	4.6	4.9	2.3	3.5	23.3
	**B5**	1.5	-	2.2	2.1	2.9	0.9	1.5	2.4	13.5
	**B4**	24.4	18.1	-	13.0	14.3	17.5	21.4	6.0	114.6
	**E1**	0.4	0.6	2.3	-	2.3	0.9	1.0	1.0	8.4
	**B3**	5.8	5.8	3.5	93.9	-	6.2	5.7	2.3	123.3
	**B2**	2.9	2.2	2.6	0.9	3.3	-	1.9	2.4	16.2
	**B1**	1.4	0.6	3.2	0.5	3.2	1.7	-	1.9	12.5
	**M1**	59.3	52.9	21.2	15.0	50.4	49.2	59.4	-	307.4
	**Sum**	95.5	83.3	37.9	127.4	81.0	81.4	93.3	19.5	-

**Figure A1:**
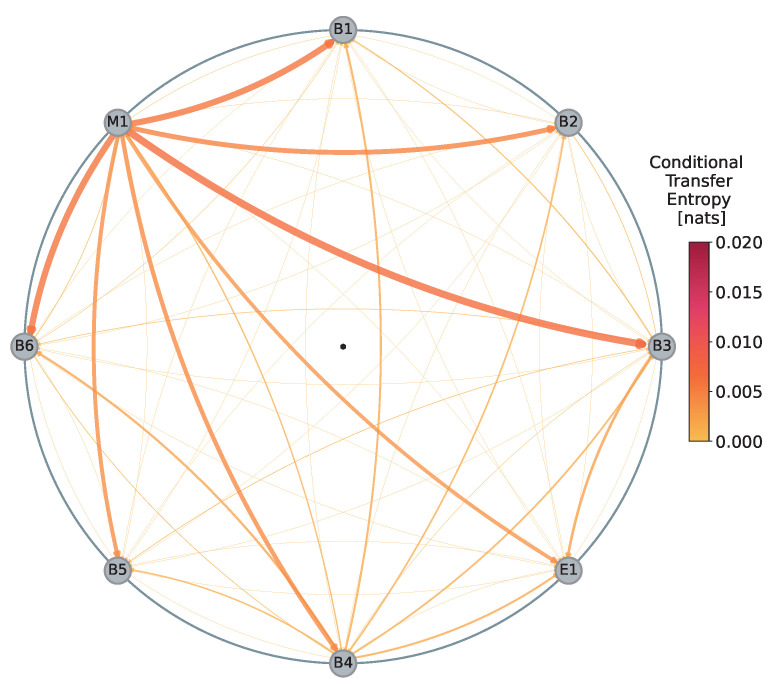
Information flow map illustrating the mean information flows in the EUR/USD dealer network for the period from 13 March to 27 March 2020. Each dealer is represented as a node, and their positions on the circle are arbitrary. The thickness and color of the directional edges between dealer pairs are proportional to the conditional transfer entropy and are expressed in nats.

**Figure A2:**
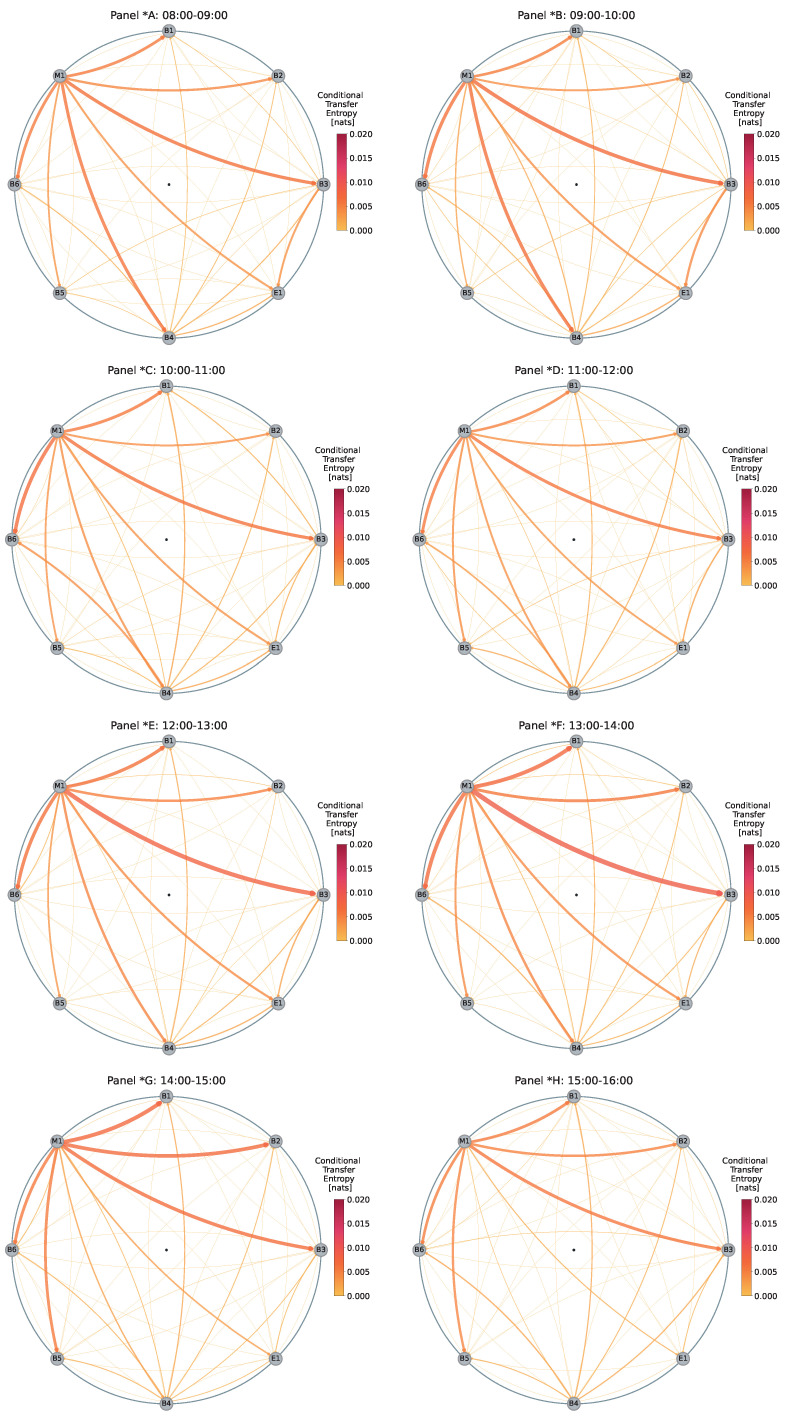
Information maps presenting the average information flows in the EUR/USD dealer network for each one-hour interval between 08:00 and 16:00 calculated using data from 11 trading days (13 March–27 March 2020).

## Data Availability

Restrictions apply to the availability of the raw data. The raw foreign exchange spot data are the property of ING Bank and are not publicly available due to privacy and legal restrictions. Intermediate data generated during the research are available from the corresponding author upon request.

## Supplementary Materials

The following supporting information can be downloaded at: https://www.mdpi.com/article/10.3390/e26090738/s1, Figure S1.1: Latency for the period from March 3 2020 till March 7 2020 in EUR/USD data set; Figure S1.2: Mean squared error of locally constant predictor for different embedding delay and dimension parameters; Figure S1.3: Flowchart illustrating the data pipeline developed for this research project; Figure S2.1: Flowchart illustrating the data pipeline developed for this research project; Figure S3.1: Validation of the KSG algorithm implementation against the analytical solution for transfer entropy; Table S1.1: Table presents the availability of dealer’s FX spot rates in the EUR/USD data set. References [52,53,54,55,56,57,58,59,60,61,62,63,64,65,66,67,68,69,70,71,72,73,74,75,76] are citied in the Supplementary Materials.

## Abbreviations

The following abbreviations are used in this manuscript:

TE	Transfer Entropy
CTE	Conditional Transfer Entropy
KSG	Kraskov, Stögbauer, and Grassberger
FX	Foreign Exchange Market
ECB	European Central Bank
GMT	Greenwich Mean Time

## Appendix A. Baseline and Hourly Information Maps for the Period of 13 March 2020–7 March 2020

In this appendix, we present additional information flow maps generated for the period following the ECB announcement, from 13 March 2020 to 27 March 2020, covering 11 trading days.
