# Supplementary material for: Inferring Dealer Networks in the Foreign Exchange Market Using Conditional Transfer Entropy: Analysis of a Central Bank Announcement
Source: Entropy (Basel). 2024 Aug 29;26(9):738. doi: 10.3390/e26090738 (PMC11431758; doi:10.3390/e26090738)
Supplement: Supplementary file 1 [file entropy-26-00738-s001.zip › entropy-3105006-supplementary.pdf]

Supplementary Material

Inferring Dealer Networks in the Foreign Exchange Market Using Conditional Transfer Entropy: Analysis of a Central Bank Announcement

This supplementary material is submitted to provide additional data, methodology details, and validation results that enhance the readers' understanding of the research presented in the publication.

Contents

Contents 1

List of Figures and Tables 2

S1 Methods 3
S1.1 Data preprocessing . . . 3
S1.1.1 Data description . . . 3
S1.1.2 Latency adjustment . . . 4
S1.1.3 Data sampling . . . 6
S1.2 Network inference algorithm . . . 7
S1.2.1 Kraskov, Stögbauer and Grassberger algorithm I . . . 7
S1.2.1.1 Time-series standardization . . . 10
S1.2.2 Parameter choice . . . 10
S1.2.2.1 State space reconstruction . . . 10
S1.2.2.2 Ragwitz criterion . . . 12
S1.2.2.3 Delay reconstruction . . . 14
S1.2.3 Statistical testing . . . 15
S1.3 Implementation remarks . . . 16

S2 Application 19
S2.1 The foreign exchange market . . . 19
S2.2 Model assumptions . . . 20
S2.3 Data . . . 20

S3 Metrics validation 22
S3.1 Transfer entropy . . . 22

Bibliography 24

List of Figures and Tables

List of Figures

S1.1 Latency for the period from March 3 2020 till March 7 2020 in EUR/USD data set. . . . 5
S1.2 Mean squared error of locally constant predictor for different embedding delay and dimension parameters. . . . 13
S1.3 Flowchart illustrating the data pipeline developed for this research project. . . . 17

S2.1 Average intraday EUR/USD quote frequency for trading days in the period February 27 2020 and March 27 2020. . . . 21

S3.1 Validation of the KSG algorithm implementation against the analytical solution for transfer entropy. . . . 23

List of Tables

S1.1 Table presents the availability of dealer's FX spot rates in the EUR/USD data set. . . . 4

Chapter S1

Methods

This chapter introduces data processing and parameter selection methods employed in the information-theoretic network inference algorithm. First, data exploratory and preprocessing efforts are described. In particular, the notion of time stamp synchronization is addressed, and the data sampling procedure is explained. Next, the Kraskov algorithm used to estimate transfer entropies is treated in detail. Additionally, the optimal delay embedding is introduced. The Ragwitz criterion and its role in determining the optimal parameter settings for the Kraskov algorithm is also addressed. Finally, the network inference algorithm implementation efforts and the main challenges faced are outlined.

S1.1 Data preprocessing

S1.1.1 Data description

We use high-frequency EUR/USD spot rate data set. The data set is comprised of privately collected, irregularly spaced, temporal data of FX rates from a selection of different dealers, provided to us by courtesy of ING Netherlands.

The EUR/USD data set is comprised of 109,449,980 high-frequency observations of the best[1] bid and ask prices quoted by eight parties between February 27 2020, and March 27 2020. This time period encompasses 22 trading days and 8 non-trading days (weekends)[2].

In our quantitative investigation, we are going to distinguish three types of trading parties; the market makers (M)[3], banks (B), and electronic trading platforms (E). Dealers' presence in particular data set is outlined in Table S1.1. While the data will be described in more detail later in Chapter S2, in this section the primary focus is on the considerations of data preprocessing.

Each data point is marked with two different timestamps, hence, we need to decide which one should be used for our investigation. Moreover, we also have to address the possibility that the temporal data are not correctly synchronized.

[1]At a particular point in time a, dealer can provide multiple ask and bit quotes for different volumes at different price points. The best bid and ask prices are the ones that represent the narrowest spread, or in other words, the most competitive ask and bid prices.

[2]While there is no trading on Saturday, on Sunday trading starts at 23:00 UTC+1 time. The reason for this is thoroughly explained in Section S2.3.

[3]Banks usually act as market makers as well, however, here, we are making a distinction between a bank and a non-bank market maker.

Data set		Dealers										
Currency pair	Time period	B1	B2	B3	B4	B5	B6	M1	E1	E2	E3	E4
EUR/USD	2020/02/27 - 2020/03/27	x	x	x	x	x	x	x	x			

TABLE S1.1: Table presents the availability of dealer's FX spot rates in the EUR/USD data set.

S1.1.2 Latency adjustment

In the context of the FX market, the latency is the time it takes for an electronic signal to travel from its origin to its destination (Addison et al. [2019], Hagströmer and Menkveld [2016]). Thus, latency captures the time between the action of a dealer updating their quote and the moment when other dealers observe that particular update. Nowadays, the average latencies have been reduced to a fraction of a millisecond with one millisecond being the upper bound (Menkveld [2013], Hasbrouck and Saar [2013], Chen et al. [2018]).

Latency is a crucial concept that needs to be addressed here since it may distort our perception of the actions and reactions in the dealer-network. For example, the quote update from bank B1 may travel for 10 milliseconds to the ING's server, whereas the signal from bank B2 may travel for 100 milliseconds. Consequently, based on the ING's quote reception timestamps, one could incorrectly infer that B1 made a quote update before B2, even if their quote updates were simultaneous.

As already mentioned, each data point in EUR/USD data set comes with two different time steps; the original timestamp that is assigned at the origin by the dealer who posts the quote (Timestamp 1) and the timestamp that ING sets at the moment when the quote update is received in the system (Timestamp 2). Both timestamps are collected and stored at a precision of 1 millisecond.

Since our goal in this project is to accurately uncover the flow of the information, the best choice is to use Timestamp 1. This is because Timestamp 1 reflects the quote updates from the perspective of the market participant posting that particular quote without any latency. On the other hand, Timestamp 2 would present the process of information flow from ING's perspective, as it would include the latencies mentioned above and hence distort our perception of dealers interactions.

The problem with using Timestamp 1 is that there is no way of verifying if the timestamps were assigned correctly. This is a concern since it is not uncommon for a dealer clock to be not synchronized with other clocks. Hence, given that we would like to use the timestamps assigned by other market participants at the origin, we must ensure that there are no anomalies present in the data. Since we do not have access to any other data than the one provided by ING, the only viable way to ensure latency consistency is to compare Timestamp 1 and Timestamp 2. Thus, the underlying assumption is that the ING's clock has been adequately synchronized and remained so over the entire time. Hence it will be used as a point of reference. With that established, the comparison of timestamps can be made to ensure that the difference between the two timestamps is not drastically changing over time, i.e. the latencies stay approximately constant.

In Figure S1.1 one can observe mean differences per hour between the ING's timestamp and timestamps assigned by different counter-parties in the EUR/USD data set. Thus, in essence we observe an average signal latency from ING's perspective. From the figure, it is clear that the mean difference between Timestamp 1 and Timestamp 2 is generally smaller than 100 milliseconds. The highest latency reaching close to 100 milliseconds is observed for electronic trading platform E1. Additionally, it can be observed that on March 6 2020, there is a spike in latency that is observed for all counter parties. While this spike is very clear on the figure, it's amplitude is not larger than 10 milliseconds as compared to the "base" latencies observed for each counter-party. Given that the data will be further resampled into 100 millisecond intervals, the spike will not have any impact on the results, and thus it can be disregarded.

While in this figure, we only take a closer look at one week of mean disparities between timestamps, the investigation is performed on the entire EUR/USD data set. This exploration does not reveal any

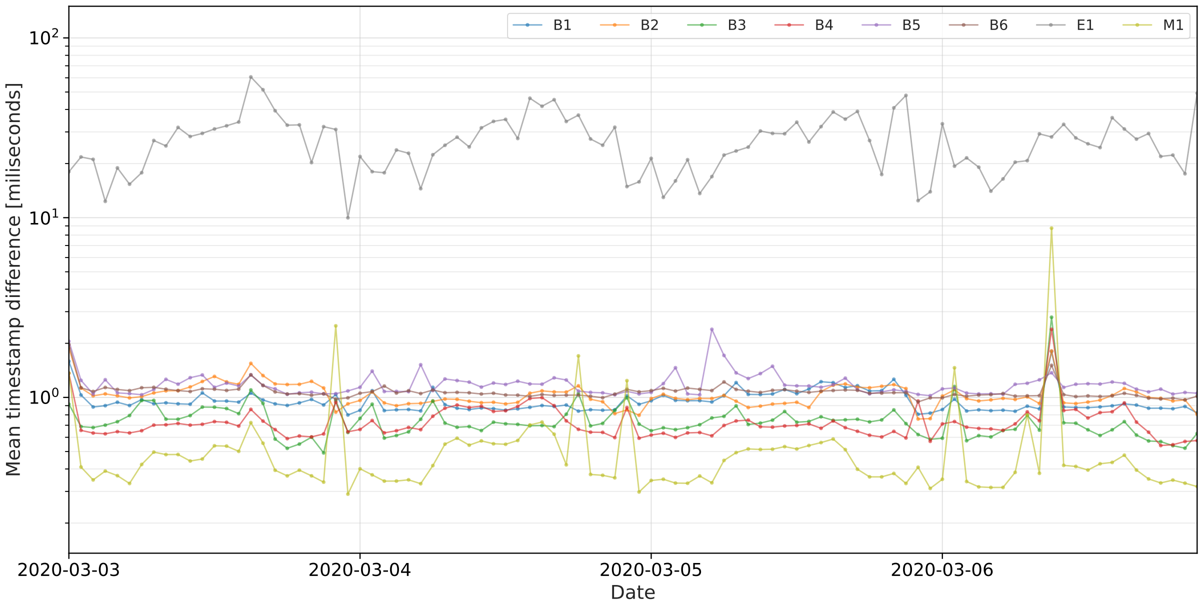

FIGURE S1.1: Mean difference between Timestamp 1 and Timestamp 2 aggregated per hour for the period from March 3 2020 till March 7 2020 in EUR/USD data set. Figure reveals how mean signal latency for various dealers fluctuates over time. The y-axis is in logarithmic scale.

anomalies in the data set. For all the counter-parties the latency remains approximately constant. Consequently, no latency adjustment is needed.

S1.1.3 Data sampling

As mentioned in the previous section, data with FX spot rates from each dealer are irregularly spaced in time simply because quote updates from dealers are posted at different times and in various time intervals between consecutive updates. Each market participant has the freedom to update its quotes whenever deemed necessary. However, to employ the information-theoretic model, the temporal data must be transformed into regularly spaced data with the same sampling frequency applied to all time series.

There are multiple reasons for that, in particular the fact that information-theoretic model relies on the data with an evenly spaced time grid. In essence, regularly spaced data amount to having a consistent number of sample points for each time series.

Many factors need to be accounted for to choose the sampling period correctly. First of all, note that the precision of the time stamps is one millisecond; hence sampling to a higher sampling period, e.g., 20 milliseconds, essentially comes down to aggregating various observations from those 20 milliseconds into one data point. Thus by undersampling, we condense the informational content of a particular series of FX rates and therefore potentially lose relevant information.

To maintain all the information that could potentially be extracted from the data, it would be best to resample the data to the lowest time interval between two quote updates of any two dealers, i.e. the precision of the clock. Only then would we be able to map all of the observations on an evenly spaced time grid. However, this approach would undoubtedly lead to a significant increase in the overall number of data points. And as it will be established in Section S1.2.1, the computational effort of the Kraskov algorithm that we will use to estimate the entropies increases as the length of the time series increases. Hence, oversampling is simply an unfeasible approach given the computational limitations.

We also need to consider the conclusions from our latency investigation. Since we are hoping to observe dealers' responses to changes in other dealers' quotes, we need to account for how the signal latency affects the reaction time of dealers to the changes in other dealers' quotes. Given that the highest mean latency observed on Figure S1.1 is close to 100 milliseconds, it is reasonable to use it as our sampling period. Assuming that other dealers do not experience significantly higher latencies than the ones we observed, 100 milliseconds is sufficient time for all dealers to observe any other dealers' updates and take action.

Finally, the so-called forward-fill approach is applied to resample the data. In simple terms, in the forward approach we fill the missing values with the closest value directly prior the missing one. This is approach is employed to ensure that no information from the future is propagated backwards in time.

S1.2 Network inference algorithm

In the network inference algorithm, both transfer entropy and conditional transfer entropy will be employed to infer information flows in the FX dealer-network. First, transfer entropy will be computed to identify dealers that are causal information contributors and quantify their information contributions for each unique dealer pair in the FX dealer-network. Next, the conditional transfer entropy will be employed to filter out the information flows that do not contribute any unique information to the target dealer, when other information contributions are considered. While it may seem like estimation of apparent and conditional transfer entropies can be easily accomplished, it is a pretty complex task, especially for continuous analogs of these metrics (Kraskov et al. [2004]). In this project, the Kraskov algorithm estimator is employed, and hence it will be thoroughly introduced in the following subsection (Wibral et al. [2014]).

S1.2.1 Kraskov, Stögbauer and Grassberger algorithm I

The concept of nearest-neighbor-based estimators for Shannon entropy was already explored a long time ago; first by Dobrushin [1958], and also later by Vasicek [1976] (Hlaváčková-Schindler et al. [2007], Kraskov et al. [2004]). However, the estimators proposed by these scholars could not be extended for the estimation of mutual information (Hlaváčková-Schindler et al. [2007]). Fairly recently, Kraskov et al. [2004] introduced a new estimator, known as Kraskov, Stögbauer and Grassberger (KSG) estimator, which has quickly gained wide acceptance in the information-theoretic community (Lizier [2014]). KSG estimator is an improved adaptation of a naive Kozaczenko-Leonenko nearest-neighbor estimator for continuous data that was proposed by Kozachenko and Leonenko [1987]. KSG is currently considered to be the top of the class transfer entropy estimator extensively used in neuroscience (Lizier [2014], Lord et al. [2018]). This is mainly because, it is a relatively simple to implement, parameter-free tool, which can handle estimating mutual information in complex multi-dimensional systems (Lord et al. [2018]). The KSG algorithm was extended for conditional mutual information estimation by Frenzel and Pompe [2007].

To be precise, Kraskov et al. [2004] propose two very similar algorithms. The theory behind these two algorithm is quiet elaborate, and is not easy to comprehend. While it is not necessary to delve into the mathematical formulation and derivation of this estimators, we will try to develop a broad understanding of these algorithms.

The differential entropy of a continuous random $\mathbb{R}$-valued variable X whose probability density function $f(x)$ is:

$$h(X) \triangleq -\int_{\mathbb{R}} f(x) \log\left(f(x)\right)\, dx$$

when the integral exists (Devroye and Györfi [2021]). The integral can also be expressed in the following manner:

$$h(X) \triangleq \int_0^1 \log\left(\frac{d}{dp}F^{-1}(p)\right)\, dp \tag{S1.1}$$

Now, following the steps of Vasicek [1976], let $x_1, x_2, x_3, \ldots, x_N$ be a sample from the probability density distribution $F(x)$. Vasicek [1976] further proposes that the distribution function F can be approximated by the empirical distribution F_n using the distances between the neighboring points. To do so, the difference operator is used instead of differential operator (Vasicek [1976]). Now, if we reorder the sample points from the smallest to the largest so that $x_1 \leq x_2 \leq \ldots \leq x_N$, then the differential entropy

can be approximated by:

$$\widehat{h}(X) = \frac{1}{N}\sum_{i=1}^{N}\log\left(N\frac{x_{i+m}-x_{i-m}}{2m}\right) \quad \forall\frac{i-1}{N} < p \leq \frac{1}{N} \tag{S1.2}$$

where $i = m+1, m+2, \ldots, N-m$ and $m < \frac{N}{2}$. If $p \leq \frac{m}{N}$ then $\frac{x_{i+m}-x_1}{2m}$ and if $p > \frac{N-m}{N}$ then $\frac{x_N - x_{i-m}}{2m}$ are used instead (Vasicek [1976]). Following this line of thought, the simplest estimator based on distances for one-dimension x was formulated as:

$$\widehat{h}(X) = \frac{1}{N-1}\sum_{i=1}^{N-1}\log\left(x_{i+1}-x_i\right) - \psi(1) + \psi(N) \tag{S1.3}$$

where, $\psi\left(x\right) = \frac{\Gamma^{'}(x)}{\Gamma(x)}$ is the digamma function[4] - the derivative of the log of the gamma function (Kraskov et al. [2004], Bossomaier et al. [2016]). The problem with this formulation is that it can not be easily generalized to higher dimensions, and therefore this method is not applicable for the estimation of mutual information (Kraskov et al. [2004]). Kozachenko and Leonenko [1987] build upon this idea and address the dimensional limitations of the naive formulation by replacing the distances between sorted points with the K-th nearest-neighbor distances in d-dimensional space (Bossomaier et al. [2016]). The idea is that this allows us to approximate the probability density, since given the distance $\frac{\epsilon}{2}$ to the K-th nearest-neighbor, this implies that there are $N-K-1$ points distance further than $\frac{\epsilon}{2}$ and $K-1$ points that are at a distance smaller then $\frac{\epsilon}{2}$ (Kraskov et al. [2004]). Consequently, the probability density function can be approximated with the trinomial formula (Kraskov et al. [2004]). The estimator of differential entropy proposed by Kozachenko and Leonenko [1987] is defined as:

$$\widehat{h}(X) = \frac{d}{N}\sum_{i=1}^{N}\log\left(\epsilon_i\right) + \log\left(c_d\right) - \psi(K) + \psi(N) \tag{S1.4}$$

where, d is the dimension of variable x, ϵ is twice the distance to the K-th nearest-neighbor, and c_d denotes the volume of the d-dimensional unit ball (Kraskov et al. [2004], Berrett et al. [2019]). For example for the Chebyshev distance (maximum norm) $c_d = 1$, whereas for Euclidean distance $c_d = \pi/\Gamma(1+d/2)/2^d$ (Kraskov et al. [2004]). It is important to note that the estimator proposed by Kozachenko and Leonenko [1987] comes with some bias, as the underlying assumption is that the density remains constant in the region captured within distance ϵ (Kraskov et al. [2004]). The bias is further controlled by K, as it effectively determines the region within distance ϵ, which is assumed to maintain a constant density. The larger the K, the further neighbor is considered, the longer the distance, hence the larger the region that is assumed to have constant density. However, as presented by Kraskov et al. [2004], the estimator comes with relatively low error scaling with $\sim K/N$ or $\sim K/N\log(N/K)$.

The bias, however, becomes a problem if one considers estimating, for example, mutual information by estimating each differential entropy separately. Recall that mutual information can be expressed as the sum of the differential entropies of the marginal and joint densities, as follows:

$$I(X;Y) = h(X) + h(Y) - h(X,Y)$$

The problem is that using Kozachenko and Leonenko [1987] estimator to compute $h(X)$, $h(Y)$ $h(X,Y)$ will results in different distance scales being used for each estimate (Kraskov et al. [2004]). In particular, the distances in the joint space will be larger than the distances in the marginal spaces (Kraskov et al. [2004]). This problem has been resolved by Kraskov et al. [2004].

Kraskov et al. [2004] propose the following solution. First, to find the K_{XY}-th nearest neighbor distance ϵ_{XY} in the joint distribution (X,Y). Next, in each marginal space determine the number of nearest neighbors that are within the respective K-th nearest neighbor distance found in the joint

[4]Digamma function is the derivative of the log of the gamma function - $\Gamma(x)$ (Bossomaier et al. [2016])

space. Thus, K is different for each marginal space, but the distance scales are the same for all spaces (Bossomaier et al. [2016]). Moreover, the bias terms are consequently of the same order and likely to cancel each other out (Bossomaier et al. [2016]). This approach is possible, since Kraskov et al. [2004] observe that Kozachenko and Leonenko [1987] formula holds for any K, hence it does not need to be fixed for the estimation of differential entropies in the marginal space (Kraskov et al. [2004]).

Definition S1.1 (KSG Algorithm I - Mutual Information)**.** Consider two continuous processes X_t and Y_t with marginal probability density functions $p(x_t)$ and $p(y_t)$. Furthermore, let us denote the joint density pf X_t and Y_t with $p(x_t, y_t)$. Then, the KSG estimate of mutual information is computed with the following formula (Kraskov et al. [2004], Lord et al. [2018]):

$$\widehat{I}(X;Y)_{KSG(1)} = \psi\left(K_{XY}\right) + \psi\left(N\right) - \frac{1}{N}\sum_{i=1}^{N}\left(\psi\left(n_X+1\right) + \psi\left(n_Y+1\right)\right) \tag{S1.5}$$

$$= \psi\left(K_{XY}\right) + \psi\left(N\right) - \langle\psi\left(n_X+1\right) + \psi\left(n_Y+1\right)\rangle \tag{S1.6}$$

where, N represents the number of points (or simply data points) that are used to estimate mutual information. Moreover, $\langle\cdots\rangle$ denotes the average over all data points, and K_{XY} represents K-th nearest-neighbor that is used to determine the distances in the joint space (X, Y) (Vejmelka and Paluš [2008]). Finally, n_X and n_Y denote the number of nearest neighbors found within the distance of ϵ_{XY} in X-space and Y-space, respectively.

In conclusion, Kraskov et al. [2004] proposed a robust estimator that can be scaled up to virtually any number of random variables. While it is a standard to use Chebyshev distance (maximum norm) to compute the distances, (as it can be easy to work with in large dimensions), in fact any distance metric can be used (Kraskov et al. [2004]). In this project, the max-norm KSG algorithm is employed.

Definition S1.2 (KSG Algorithm I - Conditional Mutual Information)**.** As mentioned in the introduction to this section, KSG algorithm for mutual information estimator was further extended to conditional mutual information by Frenzel and Pompe [2007] and adapted to transfer entropy by Gómez-Herrero et al. [2015] (Lindner et al. [2011]); the estimator takes the following form:

$$\widehat{I}(X;Y|Z)_{KSG(1)} = \psi\left(K_{XYZ}\right) + \langle\psi\left(n_Z+1\right) - \psi\left(n_{XZ}+1\right) - \psi\left(n_{YZ}+1\right)\rangle \tag{S1.7}$$

This estimator can be used to estimate the transfer entropy. The KSG algorithm can be easy extended to a case where more conditionals need to be accounted for. For example the conditional transfer entropy can be computed in the following manner:

$$\widehat{I}(X;Y|Z_1,\ldots,Z_a)_{KSG(1)} = \psi\left(K_{XYZ_1\ldots Z_a}\right) + \tag{S1.8}$$

$$\langle\psi\left(n_{Z_1\ldots Z_a}+1\right) - \psi\left(n_{XZ_1\ldots Z_a}+1\right) - \psi\left(n_{YZ_1\ldots Z_a}+1\right)\rangle \tag{S1.9}$$

where a denotes the number of information contributors that transfer entropy is conditioned on. Thus, it becomes clear how easy it is to extend the estimator to any number of conditionals.

The only parameter that one needs to specify for the KSG estimator is the K, i.e. which K-th nearest neighbor is chosen to determine the distances in the joint space that will be mapped to the marginal spaces. As mentioned earlier, K does effectively control the bias of the estimation, which here scales with a factor K/N (Kraskov et al. [2004]). However, numerical experiments strongly suggest that the estimator is relatively stable to the choice of K (of course given that $N >> K$) (Lizier [2014]). Accordingly, one must ensure that there is enough data that can be used to approximate the probability density functions, and hence estimate information-theoretic metrics.

It appears that typically K is set to 4 as a default parameter value, however it is recommended for each application to independently scan through K space to determine the appropriate choice of that parameter given the underlying distribution of the data (Lizier [2014], Wibral et al. [2014], Hlaváčková-Schindler et al. [2007]).

For example, Kraskov et al. [2004] propose to use $K = [2, 4]$, the numerical experiments presented by Frenzel and Pompe [2007] reveal that it is necessary to determine an optimal balance between systematic and statistical errors. The numerical experiments performed by Frenzel and Pompe [2007] provide a lot of important insights, in particular the fact that the highest standard error reduction is observed between $K = 2$ and $K = 8$, while the bias essentially remains the same. Similar results are presented by Runge [2014] who concludes that as we increase K the decrease in variance is much more significant than the increase in bias. The robustness of the estimator to choice of parameter K is also presented by Kraskov et al. [2004], Frenzel and Pompe [2007] and Vejmelka and Paluš [2008].

Based on the results from the literature and numerical experiments with the FX data, the decision was made to choose $K = 8$ which very significantly reduces the variance of the estimate, while maintaining negligible systematic errors. This choice was motivated by large variance of transfer entropy estimates for $K = 4$.

Finally, it is necessary to note that the choice of K parameter value also impacts the computational complexity of the nearest-neighbor searching algorithm. Higher values of K significantly extend the run time. Hence, it is necessary to balance the systematic and statistical errors, given the computational limitations. Additionally, the computational effort of the KSG algorithm also increases as the length of the time-series increases. As we have more data points, there are more data points that we need to find the nearest-neighbors for. Thus, longer time-series (N) also considerably extends the run time of the KSG algorithm.

S1.2.1.1 Time-series standardization

Apart from transforming data into stationary time-series, Kraskov et al. [2004] suggest to standardize the time series to zero mean and unit variance; this approach is widely accepted by the scientific community (Lizier [2014], Vejmelka and Paluš [2008]). After the time series is standardized, it is also recommended to introduce very low-amplitude noise to the data. For double-precision floating-point operations, Kraskov et al. [2004] proposes to add noise of order 10^{-10}. This treatment is essential when one works with empirical data with limited precision, potentially resulting in many points having equal values. Consequently, this would lead to breaking the assumption of continuously distributed points and result in spurious estimates (Kraskov et al. [2004]). Adding noise to the time series introduces very slight stochasticity to the estimator. Generally, if the information transfer is significant, it will remain significant after the addition of the noise (Lizier [2015]). The noise does not affect the estimation unless many data points have the same value. In this case, however, this would suggest that the data are not continuously distributed, and hence KSG algorithm should not be used.

S1.2.2 Parameter choice

While the KSG estimator is practically parameter-free (besides the K parameter), it is still necessary to consider the history lengths and the time-delay embedding of the time-series used with the KSG estimator. This is necessary because the past states of the stationary Markov processes can contribute significant information about their potential future state. Hence, ideally one would like to account for the entire history of the target process to properly reflect the notion of information transfer (Lizier and Rubinov [2012]). Unfortunately, in practice this is not possible with the KSG algorithm because each extra length of history introduces one more dimension that needs to be taken into account in the nearest-neighbor search. Not only does this considerably increase the computational effort, but it also exhausts the statistical power of the KSG algorithm (Lizier [2022]).

S1.2.2.1 State space reconstruction

To determine appropriate history lengths for both source and target processes and their respective time-delay embeddings, the notion of state-space reconstruction and Takens' delay embedding theorem need to be introduced.

To illustrate the idea of state-space reconstruction, consider an example of a simple pendulum, with horizontal position $x = 0$ being the center when the pendulum is at rest, $x < 0$ left-hand-side and $x > 0$ right-hand-side. Let us further assume that we do not know the length of the pendulum rod or the angle at which the pendulum was initiated. Now, if we know the position value of the pendulum at one point in time t, say $x_t = -1$, while we know that the pendulum is positioned on the left-hand side, it is impossible to tell if, at this moment of time, the pendulum is swinging to the right or left (Wibral et al. [2014]). Consequently, we have no information that could help predict its future position x_{t+1}, other than the fact that it will be in some undefined vicinity of $x \sim -1$. However, if we learn that at $t = t - 1$ the pendulum is at $x_{t-1} = -0.9$, then with that knowledge, we can reason that the pendulum is most likely still swinging towards the left. Thus, it is likely that the future position of the pendulum will be $x_{t+1} < -1$. In this manner, with a more extended history of the pendulum's position, we can determine the state that the pendulum is currently found at, e.g. broadly it is in the state of swinging to the left-hand side or on the left-hand side but swinging to the right-hand side. Thus, with a longer history of the past states, we can come up with a much better prediction of the future position of the pendulum.

However, the entire history of the pendulum may not necessarily be essential to account for. In a simple case scenario, with no gravity or any other external force in place, the pendulum's motion would be perfectly periodic. Hence, having the information collected in one full swing would provide all the information about the system. However, in a more complex scenario, the position value from time point $t = t - 100$ may be entirely irrelevant for the current dynamics, for example if some extra force was applied at $t = t - 10$. This is important to note, especially when one considers complex non-linear systems, for which we usually do not know the underlying system dynamics. The problem becomes even more involved when one needs to reconstruct the system dynamics based on just a single time-series (Bossomaier et al. [2016]).

In a nutshell, Takens [1981] proposed a theorem that the past observations of the process can be used to reconstruct the state of a d-dimensional nonlinear dynamical system (Bossomaier et al. [2016]). Let y_t denote the dependent variable observed at some discrete time points $t \in \{1, 2, \ldots, T\}$. Now, let us further assume that y_t is a function of some d-dimensional state space $\boldsymbol{x}_t = \{x_t^1, x_t^2, \ldots, x_t^d\}$, thus

$$y_t = f(\boldsymbol{x}_t) \tag{S1.10}$$

As already mentioned, while working with empirical data that represent the time-evolution of some nonlinear complex system, the underlying state-space may be unknown, or very difficult to reconstruct (Bossomaier et al. [2016]). By Takens' theorem we can reconstruct the state space from the observations of y_t, as follows:

$$\widehat{y_t} = \{y_{t-d_y\tau_{\text{emb}}}, y_{t-(d_y-1)\tau_{\text{emb}}}, y_{t-(d_y-2)\tau_{\text{emb}}} \cdots, y_{t-1}\} \tag{S1.11}$$

where τ_{emb} is called the embedding delay, which simply determines the time lag between the consecutive past states that are accounted for in the vector of past states. Also, d_y denotes the embedding dimension which is simply the length of the history y_t that is accounted for. Recall the definition of transfer entropy:

$$\text{TE}_{X_t \to Y_t}^{(d_y, d_x)} \triangleq D\left(f(y_{t+1}|\boldsymbol{y}_t^{(d_y)}, \boldsymbol{x}_t^{(d_x)})||f(y_{t+1}|\boldsymbol{y}_t^{(d_y)})\right) \tag{S1.12}$$

thus here,

$$\boldsymbol{y}_t^{(d_y)} = \{y_t, y_{t-1\tau_{\text{emb}}}, y_{t-2\tau_{\text{emb}}}, \ldots, y_{t-(d_y-1)\tau_{\text{emb}}}\} \tag{S1.13}$$

$$\boldsymbol{x}_t^{(d_x)} = \{x_t, x_{t-1\tau_{\text{emb}}}, x_{t-2\tau_{\text{emb}}}, \ldots, x_{t-(d_x-1)\tau_{\text{emb}}}\} \tag{S1.14}$$

Given the time series of length N, the number of embedding vectors that can be constructed is $N - (d_y - 1)\tau_{\text{emb}}$ (Hegger et al. [1999]). Thus, the history length and the embedding delay have a direct impact on the length of the time series that can be used for estimation with KSG algorithm.

Having introduced the Takens' theorem and elaborated on the intuition behind state-space reconstruction, we can now look into optimizing the history length and embedding delay for a give time series.

S1.2.2.2 Ragwitz criterion

Various methods exist to determine an optimal choice of embedding dimension and embedding delays. Broadly, there are two approaches: the uniform (UE) and non-uniform embedding (NUE) (Montalto et al. [2014]). The uniform embedding amounts to selecting a particular combination of embedding dimension and delay parameters for the entire time series before the process of estimating the transfer entropy between variables (Montalto et al. [2014]). On the other hand, the non-uniform embedding approach consists of progressively and individually, for each time step, selecting the most relevant past states (given some maximal lag considered) that maximizes the amount of information about the target variable (Montalto et al. [2014]). The superiority of the NUE approach over the UE lies in the fact that the NUE approach allows to minimize the dimensionality of the embedding vectors and hence improve the performance of the KSG estimator (Shahsavari Baboukani et al. [2020], Lizier [2022]). Consequently, the NUE approach becomes much more applicable when the transfer of information between a large number of processes is investigated (Novelli et al. [2019]. Since uniform embedding is a lot easier to implement, it is currently the most widely used approach (Lindner et al. [2011]). However, nowadays, much attention is given to non-uniform methods that can be tailored toward particular applications.

The decision was made to focus on the most common uniform embedding approaches, the Cao criterion Cao [1997] and Ragwitz criterion Ragwitz and Kantz [2002] (Lindner et al. [2011]). The Cao criterion is a preferred method when the optimal embedding dimension and delay need to be determined for a deterministic (chaotic) system (Lindner et al. [2011]). The Cao criterion is considered to be rather a more heuristic approach, in which the embedding dimension is determined using a false neighbor criterion, whereas the embedding delay is set to the first zero of the auto-correlation function (ACF) or the auto-information (Lindner et al. [2011], Guo et al. [2021]). As Cao [1997] states himself, his criterion is meant to provide a practical method to determine the minimum embedding dimension that is computationally efficient, does not involve any subjective parameters, and is suitable to use for time-series with high-dimensional attractors.

On the other hand, for stochastically driven systems, the Ragwitz criterion should be used instead (Lindner et al. [2011]). Ragwitz criterion provides means to jointly optimizes both the embedding dimension and delay parameters and was designed to be used for both deterministic and stochastic data from Markovian processes (Lindner et al. [2011]). Ragwitz criterion employs a locally constant predictor of the future state (w_{t+1}) of embedding vector $\boldsymbol{w}_t^k$. The prediction of the future state of the variable is estimated using the future states of the nearest neighbors of the variable after embedding is applied (Lindner et al. [2011]).

Definition S1.3 (Ragwitz's locally constant predictor)**.** Let ϵ denote the neighborhood diameter and $\mathcal{U}_n$ be the neighborhood (subscript n) of $\boldsymbol{w}_t^k$, where $\mathcal{U}_n = \{\boldsymbol{w}_n^k : ||\boldsymbol{w}_n^k - \boldsymbol{w}_t^k|| \leq \epsilon\}$. Ragwitz's locally constant estimate $\widehat{w}_{t+1}$ of the future state w_{t+1} is defined as:

$$\widehat{w}_{t+1} = \frac{1}{|\mathcal{U}_n|} \sum_{\boldsymbol{w}_n^k \in \mathcal{U}_n} y_{n+1} \tag{S1.15}$$

which simply amounts to taking the mean value of the future states (w_{n+1}) of the nearest neighbors of the embedding vector $\boldsymbol{w}_t^k$. In practice, ϵ is replaced by a specific number of K nearest neighbors taken into account for the estimation, which is a "natural" substitute when the KSG algorithm is employed (Lizier [2014]). Next, the squared error of the local predictor is computed for each time index in the embedding vector, and the mean squared error is determined.

$$e^2 = \frac{1}{|t|} \sum_t (\widehat{w}_{t+1} - w_{t+1})^2 \tag{S1.16}$$

This procedure is adopted for each combination of parameters k and τ_{emb} that one chooses to investigate. Thus, for each combination of k, and τ_{emb} the mean squared errors are determined. Based on Ragwitz's criterion, the parameters that yield the smallest mean squared error should be used.

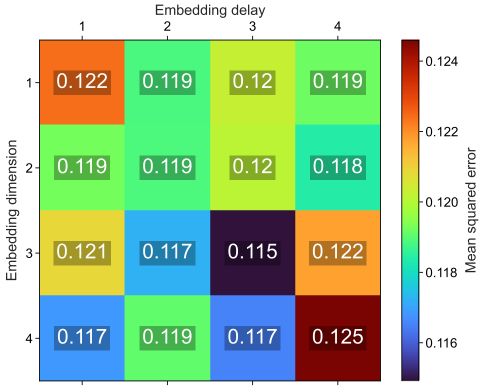

FIGURE S1.2: Mean squared error of locally constant predictor for different embedding delay and dimension parameters. The figure illustrates the parameters space that is investigated during the embedding optimization process. In this example the lowest mean squared error of 0.115 is observed for embedding delay and dimension set to 3.

Ragwitz's criterion is applied to each pair of source (X_t) and target (Y_t) processes investigated. Therefore, it is also necessary to emphasize slight but notable differences when the optimal embedding parameters are determined for source and target processes. Namely, when one investigates the optimal embedding settings for a target process Y_t, the target's future states y_{t+1} of the nearest neighbors are used for the locally constant prediction. In this setting, the embedding vector is meant to capture the underlying state of the target process for the Markov process of order d_y - hence the idea is to maximize the self-prediction (Lizier [2014]). Whereas, in the case when we want to determine the optimal embedding for the source process X_t, the target's future states y_{t+1} that correspond to the source's nearest neighbors "current" states are used instead. In other words, for each source's nearest neighbor, its corresponding future state of the target process is used in the prediction.

The reason why this is the case is very simple, namely, since we are interested in determining the transfer entropy from source to target, we are interested in the predictive power of the sources past states on the future states of the target. Thus, naturally we are interested in finding the optimal embedding that minimizes the prediction of the future states of the target process.

Moreover, it is necessary to emphasize the importance of the proper state-space reconstruction. As already discussed when the notion of transfer entropy was introduced, the history of the target process, especially when one considers Markovian process, may in fact provide a lot of significant information about its future state (Lizier [2014]). Hence, given that we cannot include the entire history of the target process, and thus we are making some approximations, it is necessary to perform the state-space reconstruction with utmost attention. As insufficient state-space reconstruction may lead to spurious conclusions about the flow of information from one variable to another, when in fact it is the history of the target process that contributes the information (Wibral et al. [2014]).

Finally, it should be noted that the state space reconstruction and the Ragwitz criterion, which we use for the nonlinear models, is an approach analogous to using the Bayesian information criterion for the linear models (Pan and Duraisamy [2020]). In essence, both criterion are used to determine the length of the history of the variable that needs to be accounted for.

In our information-theoretic network inference algorithm, the maximal embedding dimension and delays are set to 4. Higher embedding dimensions would be computationally unfeasible given the scope of this research project and computational limitations of the KSG algorithm, which we elaborated on in this section.

S1.2.2.3 Delay reconstruction

After the embedding dimension and delays of both source and target processes are determined, it is necessary to determine the "true" delay for the source-target interaction that is investigated Wibral et al. [2012]. Up to this point we have not explicitly considered the possibility of the information being transferred at any other delay than delay of 1. In practice, when we work on complex systems, we do not have much or any knowledge about the underlying system dynamics. Thus, it may not always be possible to know what is the "true" delay between the interaction of source and target processes.

Wibral et al. [2013] not only provides the method to determine an optimal delay that should be used for the estimation of transfer entropy, but also they prove that the optimal delay is in fact the true delay. Thus, even if one works with the data that was not correctly adjusted for the latency, the transfer entropy will still be detected, just at a different delay.

The method for delay reconstruction as proposed by Wibral et al. [2013] and also suggested by Lizier [2022] is in fact very simple. In order to determine the optimal delay between source and target processes it is necessary to estimate transfer entropy for each delay in the delay-space that one considers. The true source-target delay is the one that yields the largest transfer entropy estimate. The transfer entropy is defined as:

$$\mathrm{TE}_{X_t \to Y_t}^{(d_y,d_x)} = I(Y_{t+1}; \boldsymbol{X}_t^{(d_x)} | \boldsymbol{Y}_t^{(d_y)}) \tag{S1.17}$$

for the purpose of discussion of the source-target delay note that we can also represent it slightly differently, as follows:

$$\mathrm{TE}_{X_t \to Y_t}^{(d_y,d_x)} = I(Y_t; \boldsymbol{X}_{t-u}^{(d_x)} | \boldsymbol{Y}_{t-1}^{(d_y)}) \tag{S1.18}$$

by simply shifting future and history data points of Y_t process by one backwards, and introducing the delay parameter u for the source process X_t. In this formulation u represents source-target delay. Given this formulation, the optimal source-target delay is determind to be:

$$\delta = \underset{u \in \mathcal{U}}{\operatorname{argmax}} \left(I(Y_t; \boldsymbol{X}_{t-u}^{(d_x)} | \boldsymbol{Y}_{t-1}^{(d_y)}) \right) \tag{S1.19}$$

where $\mathcal{U}$ is the investigated delay-space, i.e. $\mathcal{U} = \{1, 2, 3, \ldots, u_{\max}\}$, and δ represent the true delay between source and target processes.

Note that we only consider different delays between the source and target processes. The delay between the target's history and the target's future states needs to remain equal to one. Changing

the delay between the target's future states and the target's history would violate Wiener's principle of causality (Wibral et al. [2013])[5].

Finally, Wibral et al. [2013] extends upon the notion of "true" delay by providing the framework for detection of multiple delays and feedback loops. While this is very interesting, it needs to remain as potential future extension of this research project.

In our information-theoretic network inference algorithm, we set the maximal source-target delay to 4 (which, given the resampling interval, is equivalent to 400 ms). From our latency investigation and the literature, it is clear that 400ms is more than enough time for dealers to react to quote updates of other dealers. Furthermore, the analysis of the distribution of true source-target delays revealed that most of the true delays recovered were equal to 1.

S1.2.3 Statistical testing

Having estimated the transfer entropy between the source and target processes, it is necessary to assess whether the estimate is statistically significant. While in theory, the estimate of transfer entropy should be zero if the future state of the target process is conditionally independent from its own history and the history of the source process, this is not exactly the case when the KSG algorithm is used.

As previously stated, not only does the KSG algorithm introduce bias, but also we are working with finite-size sample and we introduce low-amplitude noise to the data. Hence, the transfer entropy estimate will not necessarily be equal to zero, even if the source's history does not provide unique information about the target's future state. Therefore, it is imperative to assess the statistical significance of transfer entropy estimates.

Since we can not make any assumptions about the underlying distribution of transfer entropy estimates, we need to employ alternative techniques such as permutation testing or bootstrapping (Bossomaier et al. [2016], Wibral et al. [2012], Lizier [2014]). The permutation testing is currently the most common method to assess statistical significance of information theoretic estimates (see Lindner et al. [2011], Lizier [2014], Wibral et al. [2013], Wollstadt et al. [2018]), hence it is also employed in this research project.

When performing the statistical assessment with permutation technique, we are essentially assessing whether the estimated KL divergence between transitional probabilities $p(y_{t+1}|\boldsymbol{y}_t^{(d_y)})$ and $p(y_{t+1}|\boldsymbol{y}_t^{(d_y)}, \boldsymbol{x}_t^{(d_x)})$ is indeed statistically significant. Consequently, we are assessing if the information provided by the source process does in fact contribute unique information about the future state of the target process. In practice, we are interested in testing for the null hypothesis that the state changes $\boldsymbol{y}_t^{(d_y)} \rightarrow y_{t+1}$ have no temporal dependence on the source process $\boldsymbol{x}_t^{(d_x)}$ (Lizier et al. [2011]), hence:

$$\mathbf{H_0} : p(y_{t+1}|\boldsymbol{y}_t^{(d_y)}) = p(y_{t+1}|\boldsymbol{y}_t^{(d_y)}, \boldsymbol{x}_t^{(d_x)}) \implies \widehat{\mathrm{TE}}_{X_t \rightarrow Y_t}^{(d_y,d_x)} = 0 \tag{S1.20}$$

$$\mathbf{H_1} : p(y_{t+1}|\boldsymbol{y}_t^{(d_y)}) \neq p(y_{t+1}|\boldsymbol{y}_t^{(d_y)}, \boldsymbol{x}_t^{(d_x)}) \implies \widehat{\mathrm{TE}}_{X_t \rightarrow Y_t}^{(d_y,d_x)} > 0 \tag{S1.21}$$

The one-sided alternative hypothesis is motivated by the fact that transfer entropy is a non-negative measure. To test the above-presented hypotheses, we need to generate an empirical distribution of transfer entropy estimates under null hypothesis. This can be done by generating a large number of source process surrogates (X_t^s), which preserve transitional probability $p(y_{t+1}|\boldsymbol{y}_t^{(d_y)})$, but destroy the dependence in $p(y_{t+1}|\boldsymbol{y}_t^{(d_y)}, \boldsymbol{x}_t^{(d_x)})$ (Lizier [2014]). While the surrogates can be generated in many different ways, the most important aspect is to ensure that the vectors of past states of source process $\boldsymbol{x}_t^{(d_x)}$ are preserved (unless $d_x = 1$). This is crucial, because we need to retain the reconstructed state-spaces (Lizier [2014]). Thus, the easiest way to generate the surrogates would be shuffling the vectors of past states $\boldsymbol{x}_t^{(d_x)}$ among the set of $\{y_{t+1}, \boldsymbol{y}_t^{(d_y)}, \boldsymbol{x}_t^{(d_x)}\}$ tuples - i.e. interchanging their time indices within the time-series (Lizier [2014]). This approach is also employed in this research project.

[5] An interested reader is referred to Wibral et al. [2013], where the reason behind not delaying target's history and the notion of self prediction optimality are clearly explained.

Let us assume that we generated S of source process surrogates (X_t^s). Then, the p-value is determined by simply counting the number of cases when $\widehat{\text{TE}}^{(d_y,d_x)}_{X_t^s \to Y_t} > \widehat{\text{TE}}^{(d_y,d_x)}_{X_t \to Y_t}$. In other words, if the transfer entropy estimated for a surrogate source process is greater than the actual transfer entropy estimated for the original source process, then this suggests that our transfer entropy estimate is not significant. This is because, such scenarios would indicate that even if we destroy the temporal precedence structure our estimator still can yield higher value of transfer entropy (Wibral et al. [2014]), which undermines the significance of the original estimate. Thus, the p-value is determined in the following manner:

$$\text{p-value} = \frac{1}{S}\sum_{i=1}^{S} \mathbb{1}\left(\widehat{\text{TE}}_{i\ X_t^s \to Y_t}^{(d_y,d_x)} > \widehat{\text{TE}}_{X_t \to Y_t}^{(d_y,d_x)}\right) \tag{S1.22}$$

where $\mathbb{1}(\cdot)$ denotes the indicator function, and S is the number of surrogate source processes. For a given significance level α, we reject $\mathbf{H_0}$ if p-value $< \alpha$ (Lizier et al. [2011]). Additionally, one should note that since we are going to conduct multiple hypothesis tests, we need to employ Bonferroni correction.

The decision on how many permutations are needed to provide a robust p-value is not straightforward. Of course, the more permutations performed, the more robust the computed p-value. However, in practice we are restricted by the computational limitations since estimating the transfer entropy for each surrogate of the source process is computationally equivalent to estimating a transfer entropy for the original source process. Hence, while we should investigate the robustness of the p-value to the number of permutations performed, in our information-theoretic network inference algorithm the number of permutations is set to $S = 500$. S is set to 500 because this is the largest number of permutations that could be performed within the time limits of this research project. Note that we are expecting to compute a total of 107,520 entropy estimates. Thus, if we perform 500 permutations for each estimate, we are looking at computing approximately up to 54 million entropy estimations, which is already quite a large number.

Finally, one should note that $S = 500$ is not a low number of permutations; for example, Lizier et al. [2011] use only $S = 300$ to assess the statistical significance of their estimates. As discussed later in the results section, only a very small portion of statistically significant apparent or conditional transfer entropies are found to have a p-value greater than zero.

S1.3 Implementation remarks

Since the network inference algorithm is employed on an extensive data set, a data pipeline had to be developed. The pipeline in Figure S1.3 is developed using three programming languages; `Python` and `C++` wrapped together with `Cython`. The transition between `Python` and `C++` via `Cython` takes place on the verges of purple box, where modules within the box were developed in `C++`. All of the modules are compiled together as one `Python` library. As presented in Figure S1.3, the developed pipeline is comprised of multiple modules that perform various tasks from data transformation to analysis with an network inference algorithm.

The pipeline starts with the data for a particular day of the week getting fetched from the Amazon Web Services (AWS) S3 bucket. In the data transformation module, raw data are first transformed from a `qtable` format to `Pandas DataFrame`. Next, from the `DataFrame` columns with relevant information and the best bid-and-ask prices are extracted. For example, the choice can be made to use Timestamp 1 instead of Timestamp 2. Next, the data is cleaned from observations that have missing entries in any remaining columns. The number of removed and remaining data observations is recorded and later reviewed to ensure the integrity of the data, data cleaning process, and the subsequent analysis.

In the preprocessing module, the data is first resampled to a defined sampling frequency, followed by data extraction for chosen hours of the day, e.g., between 8:00 and 16:00. Afterwards, the time series is differenced to ensure stationarity and split into five-minute-long subsamples fed the network inference algorithm. Effectively, an 8-hours-long time window yields 96 five-minute-long subsamples.

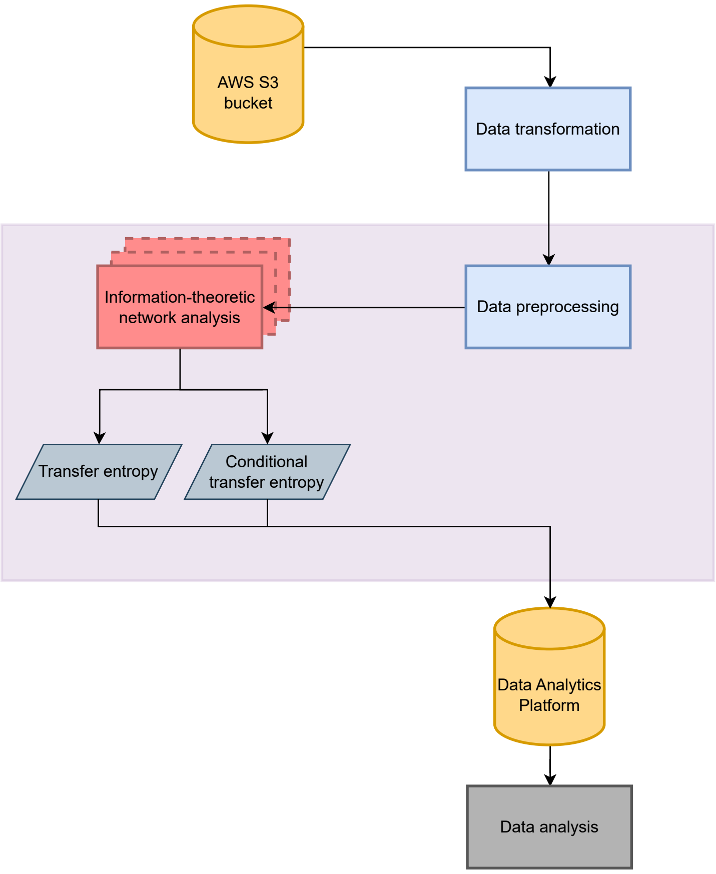

FIGURE S1.3: Flowchart illustrating the data pipeline developed for this research project.

In addition to being differenced the data is also standardized, and a low-amplitude noise is added. The network inference algorithm is also parallelized, where each CPU performs analysis on different subsamples from a particular day. The data produced from the network inference algorithm are then merged and saved as multidimensional `NumPy` matrices into the Data Analytics Platform's S3-backed filesystem. Further data analysis-related activities are performed on this platform.

The network inference algorithm is very computationally demanding. On average, it takes 24 hours to perform the information-theoretic analysis on eight hours' worth of data[6]. Consequently, it takes the entire pipeline about a 2 weeks to process all of the EUR/USD data in the scope of this project.

While a month-long run time may appear lengthy, it is expected, given that the KSG algorithm is very computationally demanding. As discussed in Section S1.2.1, for each transfer and conditional transfer entropy, we need to perform four multidimensional nearest-neighbors searches. To be exact, one nearest neighbor search involves determining distances to the Kth nearest neighbors of each data point in the joint space, and the other three need to determine the number of nearest neighbors that can be found within these distances in the other joint and marginal spaces. Moreover, the nearest neighbor search is also employed in the algorithm that determines the optimal dimension and delay embedding for each source-target pair. Finally, the whole process is repeated for each surrogate used for permutation testing. In practice, however, the algorithm is optimized by aborting the permutation testing when the p-value of the estimate exceeds the predefined minimum significance level required.

A naive algorithm for the nearest neighbors search using Chebyshev distances scales with $O(DN^2)$, where D is the dimension of the data, and N is the number of data points (Pedregosa et al. [2011], Lizier [2014]). Note that for a joint space, the dimension D is equivalent to the sum of embedding dimensions

[6]For a network of 8 dealers with the time series of quotes resampled to 100 milliseconds, using 8 CPU cores and 500 permutations per entropy estimate. Moreover, the maximal embedding dimension is set to 4.

of all variables. Hence the dimension can get quite large. For fast nearest neighbor algorithm such as KD-tree, the nearest neighbors search complexity scales with $O(DN \log N)$ (Pedregosa et al. [2011], Lizier [2014]).

The performance of the KD-tree is the bottleneck of the network inference algorithm; therefore, much attention was put into developing a high-performance KD-tree algorithm. Not only was the algorithm first developed from scratch, but also many openly available `C++` implementations were tested. None of the algorithms were able to perform fast enough to complete the analysis of such a large data set within the time limits of this research project. Finally, it was determined that `Scipy's` implementation of KD-tree in `Python` was outperforming any implementation in `C++`. The source code for this implementation turned out to be also written in `C++`. Hence, `Scipy's` source code was adjusted, compiled as a separate dynamic library, and used in the network inference algorithm (Virtanen et al. [2020]). As a result of this change, the KD-tree nearest neighbor search for 3000 data points in 1D takes as little as 70 milliseconds. Consequently, the performance KSG algorithm for transfer entropy was able to significantly outperform[7] the implementation from JIDT Libary (Lizier [2014]). Thus, it was the most significant breakthrough that made it possible to meet the research project goals and perform network inference investigation on a large data set.

[7] Not considering the usage of GPUs.

Chapter S2

Application

S2.1 The foreign exchange market

The foreign exchange market is a worldwide, decentralized marketplace that facilitates trading of currencies between various market participants. Over the past years, we have observed a significant change in the structure of the FX market with the emergence of new types of market players (King et al. [2011]). Since in this research project we attempt to quantify the information flows between various market participants, it is necessary to outline the structure of the FX market, explain how it operates, and introduce the types of market participants involved in the market. This is necessary to provide the reader with a general overview of how information is created and flows through the market as well as establish the differences in customers that each dealer caters to.

The key market participants who naturally emerge in the FX market are the so-called market makers, broadly referred to as dealers. For a large part, banks perform the role of market makers; however, they are not the only market participants. In this research project, one non-bank market maker is considered. Market makers' primary function is to provide liquidity to the market, which means they are ready to trade with anyone at any time. They facilitate the trading process by allowing clients to quickly make trades without the need to wait for a counter-party that would match the trade on the other side. Additionally, they contribute toward maintaining competitive bid-ask rates on the market. In the FX market, dealers not only trade with each other but also cater to their private clients. Such clients could be, for example, a multinational company or importer/exporter operating in Euro and occasionally making a deal in US dollars.

Now, let us consider how the FX market operates. Clients approach a dealer, for example through their bank, which quotes the bid and ask the prices based on their clients' need. Clients can complete the transaction or search for a better quote from a different dealer. When the client's order is executed, the FX dealer trades in the inter-dealer market to unwind his customer's trade (Vitale [2006]). Trading on the inter-dealer market can take place in two different ways: the dealer can approach another dealer directly or indirectly via public limit order books operated by electronic brokerage platform such as EBS. When a trade takes place directly between two dealers, such transaction is termed a bilateral or private meeting. On the other hand, on electronic brokerage platforms, subscribers can add limit orders or match outstanding ones (Vitale [2006]). When the orders of both parties are matched, only the dealers involved in the transaction know each other's identity.

In the microstructure approach, we recognize that private information in the decentralized markets is produced locally and then transferred between the dealers (Hagströmer and Menkveld [2019], Hasbrouck [1996]). Relevant private information is obtained from direct clients of each dealer. The clients observe the fundamentals and basing upon them update their views. Next, they trade with FX dealers, who observe the orders of all of their clients – the order flow. Dealers observe the order flow and accordingly set the price for the currency exchange. Finally, this information is further conveyed to other dealers, which is reflected in the exchange rates updates send by the dealer. In this manner, the information is produced

directly based on the demand of private clients and conveyed via FX dealers to the dealer-network. On the other hand, when we consider public information, the microstructure approach proposes that all public information is directly impounded into the price by dealers; however, there may exist disparities between dealers in the interpretation of the news.

S2.2 Model assumptions

In the following subsection, the underlying model assumptions are clearly stated.

- Dealers see the quotes of all other dealers in the dealer-network.
- The latency between the action of a dealer updating their quote and the moment other dealers observe that particular update is smaller than the time interval used in data re-sampling.
- The asymmetry in the information is attributed to the dealer's private information. Private information is attributed to the internal order flow observed by each dealer.
- We assume that CTE quantifies the unique transfer of information between dealers.

S2.3 Data

As mentioned in Section S1.1.1 our goal is to perform an analysis on the FX spot data for EUR/USD currency pair traded between February 27 2020 and March 27 2020.

Foreign exchange market is open 24 hours per day as it needs to cater to customers located across different time zones. However, the activity on the FX market varies over time, with dealers providing more frequent updates on the quotes on particular days of the week and at particular times of the day. Since we are going to work with a lot of data, we would like to sample the time windows for the days on which the dealers are most active.

The changes in activity on the FX market can be explained by considering the timezones for each major region. For example, on Saturday we do not observe any activity on the FX market as all of the sessions are closed, and on Sunday there is very little activity given that only the Sydney session is open. For this reason, we exclude the data from the Saturdays and Sundays from the analysis as relatively very little to no activity is observed on these days.

Next, we investigate the intraday quote frequency in the EUR/USD dataset. For each trading day in the data set, the days are split into 30-min time windows. In each time window we count the number quote updates from each dealer and then the average number of quote updates per second is determined. The intraday quote frequency aggregated per each 30-minute time windows in the day is presented in Figure S2.1. Note that the figure illustrates the average quote frequency observed cumulatively for all dealers in the data set. Thus, in order to obtain an average quote frequency per second per dealer, the values would need to be normalized by the number of the dealers.

From Figure S2.1 it is clear that changes in the activity closely align with different timezones overlaps. Let us consider the generally accepted timezones for four major sessions; New York 14:00-23:00, Tokyo 1:00-10:00, Sydney 22:00-7:00 and London 8:00-17:00 (UTC+1). In Figure S2.1 we observe a spike in activity at 8:00, at the opening of the London session, and relatively high activity prevails until an hour before end of London session.

Additionally, we can observe another spike in activity at 13:00, which is the hour of opening of the New York session. The highest average quote frequency is observed when both London and New York sessions are open. Based on the results from this investigation, we sample from time windows between 8:00 and 16:00 on consecutive days, since clearly in this time window the highest activity is observed.

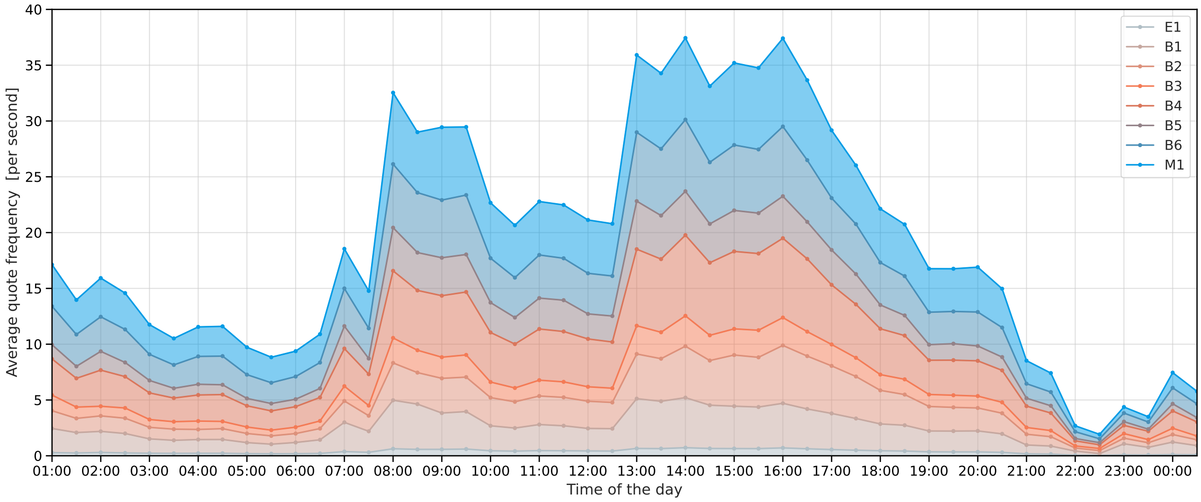

FIGURE S2.1: Average intraday EUR/USD quote frequency for trading days in the period February 27 2020 and March 27 2020. Figure illustrates how average quote frequency of all dealers in the data set evolves throughout the day. The average quote frequencies are aggregated per 30 minute time windows. For example value at 13:00 represents the quote frequency observed between 13:00 and 13:30.

Chapter S3

Metrics validation

In this chapter, we present the validation results for the transfer entropy metric, which is validated with the analytical solution for transfer entropy between two Gaussian coupled auto-regressive processes. We use the analytical solution derived by Kaiser and Schreiber [2002].

S3.1 Transfer entropy

To validate the KSG algorithm's implementation for the transfer entropy estimation, we compare our transfer entropy estimates with the analytical solution provided by Kaiser and Schreiber [2002]. The analytical solution is provided for Gaussian coupled auto-regressive processes, which need to be simulated. The following system of equations defines the coupled processes X_t and Y_t.

$$X_{t+1} = \alpha X_t + \epsilon_t \tag{S3.1}$$

$$Y_{t+1} = \beta Y_t + \gamma X_t + \omega_t \tag{S3.2}$$

where ϵ_t and ω_t are independent standard normal random variables.

Following the steps of Kaiser and Schreiber [2002] we use $\alpha = 0.5$ and $\beta = 0.6$. Next, we simulate processes X_t and Y_t for two different sample sizes; 3000 and 1 million. Additionally, the data is generated for 20 different coupling parameter values γ. And finally, the transfer entropy between the processes is estimated with our KSG algorithm and computed using the analytical solution. The comparison of the KSG estimate to the analytical solution is presented in Figure S3.1.

From Figure S3.1, it is clear that the analytical solution nearly perfectly aligns with our KSG transfer entropy estimates for a large sample size. Therefore, small disparities observed for the smaller sample size can be disregarded because they result from finite-size effects.

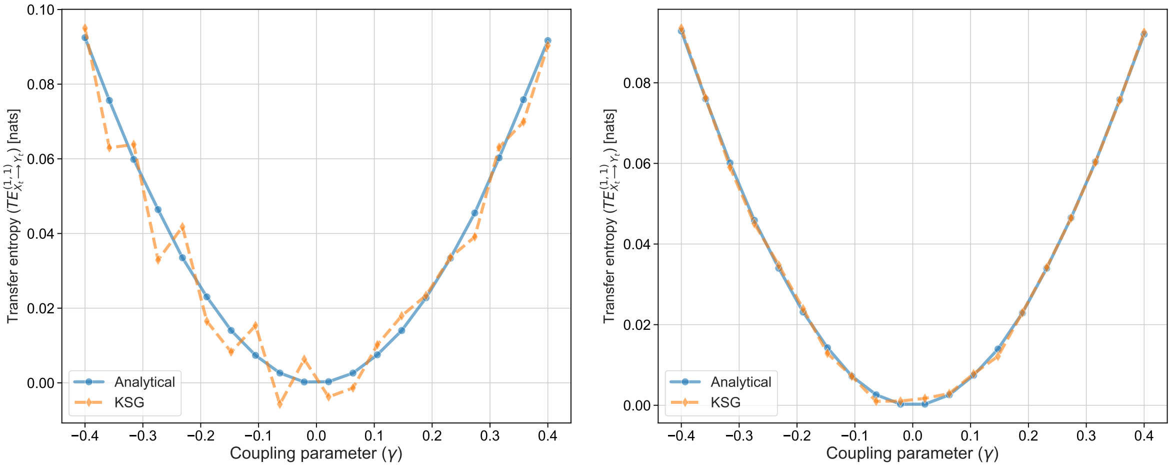

FIGURE S3.1: Validation of the KSG algorithm implementation against the analytical solution for transfer entropy. The plot on the left-hand side presents the transfer entropies estimated for a sample size of 3000. At the same time, the plot on the right-hand side compares both estimates for a sample size of 1 million data points.

Bibliography

Andrew Addison, Charles Andrews, Newas Azad, Daniel Bardsley, John Bauman, Jeffrey Diaz, Tatiana Didik, Komoliddin Fazliddin, Maria Gromoa, Ari Krish, et al. Low-latency trading in the cloud environment. In *2019 IEEE International Conference on Computational Science and Engineering (CSE) and IEEE International Conference on Embedded and Ubiquitous Computing (EUC)*, pages 272–282. IEEE, 2019.

Thomas B Berrett, Richard J Samworth, and Ming Yuan. Efficient multivariate entropy estimation via k-nearest neighbour distances. *The Annals of Statistics*, 47(1):288–318, 2019.

T Bossomaier, L Barnett, M Harré, and JT Lizier. An Introduction to Transfer Entropy: Information Flow in Complex Systems. Cham, Switzerland: Springer International Publishing; 2016, 2016.

Liangyue Cao. Practical method for determining the minimum embedding dimension of a scalar time series. *Physica D: Nonlinear Phenomena*, 110(1-2):43–50, 1997.

Shu-Heng Chen, Mak Kaboudan, and Ye-Rong Du. *The Oxford handbook of computational economics and finance*. Oxford University Press, 2018.

Luc Devroye and László Györfi. On the consistency of the Kozachenko-Leonenko entropy estimate. *IEEE Transactions on Information Theory*, 2021.

Roland L'vovich Dobrushin. A simplified method of experimentally evaluating the entropy of a stationary sequence. *Theory of Probability & Its Applications*, 3(4):428–430, 1958.

Stefan Frenzel and Bernd Pompe. Partial mutual information for coupling analysis of multivariate time series. *Physical review letters*, 99(20):204101, 2007.

Germán Gómez-Herrero, Wei Wu, Kalle Rutanen, Miguel C Soriano, Gordon Pipa, and Raul Vicente. Assessing coupling dynamics from an ensemble of time series. *Entropy*, 17(4):1958–1970, 2015.

Zhenghao Guo, Verity M McClelland, Osvaldo Simeone, Kerry R Mills, and Zoran Cvetkovic. Multiscale Wavelet Transfer Entropy with Application to Corticomuscular Coupling Analysis. *IEEE Transactions on Biomedical Engineering*, 69(2):771–782, 2021.

Björn Hagströmer and Albert J Menkveld. A network map of information percolation. *Working Paper*, 2016.

Björn Hagströmer and Albert J Menkveld. Information revelation in decentralized markets. *The Journal of Finance*, 74(6):2751–2787, 2019.

Joel Hasbrouck. 22 Modeling market microstructure time series. *Handbook of statistics*, 14:647–692, 1996.

Joel Hasbrouck and Gideon Saar. Low-latency trading. *Journal of Financial Markets*, 16(4):646–679, 2013.

Rainer Hegger, Holger Kantz, and Thomas Schreiber. Practical implementation of nonlinear time series methods: The TISEAN package. *Chaos: An Interdisciplinary Journal of Nonlinear Science*, 9(2): 413–435, 1999.

Katerina Hlaváčková-Schindler, Milan Paluš, Martin Vejmelka, and Joydeep Bhattacharya. Causality detection based on information-theoretic approaches in time series analysis. *Physics Reports*, 441(1): 1–46, 2007.

Andreas Kaiser and Thomas Schreiber. Information transfer in continuous processes. *Physica D: Nonlinear Phenomena*, 166(1-2):43–62, 2002.

Michael R King, Carol L Osler, and Dagfinn Rime. Foreign exchange market structure, players and evolution. 2011.

Lyudmyla F Kozachenko and Nikolai N Leonenko. Sample estimate of the entropy of a random vector. *Problemy Peredachi Informatsii*, 23(2):9–16, 1987.

Alexander Kraskov, Harald Stögbauer, and Peter Grassberger. Estimating mutual information. *Physical review E*, 69(6):066138, 2004.

Michael Lindner, Raul Vicente, Viola Priesemann, and Michael Wibral. TRENTOOL: A Matlab open source toolbox to analyse information flow in time series data with transfer entropy. *BMC neuroscience*, 12(1):1–22, 2011.

Joseph Lizier. Is KSG estimator deterministic? - [Java Information Dynamics Toolkit (JIDT) discussion], 2015. URL `https://groups.google.com/g/jidt-discuss/c/EmLEXo9BGcA/m/ReOAnwc6DgAJ`.

Joseph Lizier. Ragwitz auto-embedding in conditional transfer entropy - [Java Information Dynamics Toolkit (JIDT) discussion], 2022. URL `https://groups.google.com/g/jidt-discuss/c/TEcGwPQ__7U`.

Joseph Lizier and Mikail Rubinov. Multivariate construction of effective computational networks from observational data. 2012.

Joseph T Lizier. JIDT: An information-theoretic toolkit for studying the dynamics of complex systems. *Frontiers in Robotics and AI*, 1:11, 2014.

Joseph T Lizier, Jakob Heinzle, Annette Horstmann, John-Dylan Haynes, and Mikhail Prokopenko. Multivariate information-theoretic measures reveal directed information structure and task relevant changes in fMRI connectivity. *Journal of computational neuroscience*, 30(1):85–107, 2011.

Warren M Lord, Jie Sun, and Erik M Bollt. Geometric k-nearest neighbor estimation of entropy and mutual information. *Chaos: An Interdisciplinary Journal of Nonlinear Science*, 28(3):033114, 2018.

Albert J Menkveld. High frequency trading and the new market makers. *Journal of financial Markets*, 16(4):712–740, 2013.

Alessandro Montalto, Luca Faes, and Daniele Marinazzo. MuTE: a MATLAB toolbox to compare established and novel estimators of the multivariate transfer entropy. *PloS one*, 9(10):e109462, 2014.

Leonardo Novelli, Patricia Wollstadt, Pedro Mediano, Michael Wibral, and Joseph T Lizier. Large-scale directed network inference with multivariate transfer entropy and hierarchical statistical testing. *Network Neuroscience*, 3(3):827–847, 2019.

Shaowu Pan and Karthik Duraisamy. On the structure of time-delay embedding in linear models of nonlinear dynamical systems. *Chaos: An Interdisciplinary Journal of Nonlinear Science*, 30(7):073135, 2020.

F. Pedregosa, G. Varoquaux, A. Gramfort, V. Michel, B. Thirion, O. Grisel, M. Blondel, P. Prettenhofer, R. Weiss, V. Dubourg, J. Vanderplas, A. Passos, D. Cournapeau, M. Brucher, M. Perrot, and E. Duchesnay. Scikit-learn: Machine Learning in Python. *Journal of Machine Learning Research*, 12: 2825–2830, 2011.

Mario Ragwitz and Holger Kantz. Markov models from data by simple nonlinear time series predictors in delay embedding spaces. *Physical Review E*, 65(5):056201, 2002.

Jakob Runge. Detecting and quantifying causality from time series of complex systems. 2014.

Payam Shahsavari Baboukani, Carina Graversen, Emina Alickovic, and Jan Østergaard. Estimating Conditional Transfer Entropy in Time Series Using Mutual Information and Nonlinear Prediction. *Entropy*, 22(10):1124, 2020.

Floris Takens. Detecting strange attractors in turbulence. In *Dynamical systems and turbulence, Warwick 1980*, pages 366–381. Springer, 1981.

Oldrich Vasicek. A test for normality based on sample entropy. *Journal of the Royal Statistical Society: Series B (Methodological)*, 38(1):54–59, 1976.

Martin Vejmelka and Milan Paluš. Inferring the directionality of coupling with conditional mutual information. *Physical Review E*, 77(2):026214, 2008.

Pauli Virtanen, Ralf Gommers, Travis E. Oliphant, Matt Haberland, Tyler Reddy, David Cournapeau, Evgeni Burovski, Pearu Peterson, Warren Weckesser, Jonathan Bright, Stéfan J. van der Walt, Matthew Brett, Joshua Wilson, K. Jarrod Millman, Nikolay Mayorov, Andrew R. J. Nelson, Eric Jones, Robert Kern, Eric Larson, C J Carey, İlhan Polat, Yu Feng, Eric W. Moore, Jake VanderPlas, Denis Laxalde, Josef Perktold, Robert Cimrman, Ian Henriksen, E. A. Quintero, Charles R. Harris, Anne M. Archibald, Antônio H. Ribeiro, Fabian Pedregosa, Paul van Mulbregt, and SciPy 1.0 Contributors. SciPy 1.0: Fundamental Algorithms for Scientific Computing in Python. *Nature Methods*, 17:261–272, 2020. doi: 10.1038/s41592-019-0686-2.

Paolo Vitale. A market microstructure analysis of foreign exchange intervention. 2006.

Michael Wibral, Patricia Wollstadt, Ulrich Meyer, Nicolae Pampu, Viola Priesemann, and Raul Vicente. Revisiting Wiener's principle of causality—interaction-delay reconstruction using transfer entropy and multivariate analysis on delay-weighted graphs. In *2012 Annual International Conference of the IEEE Engineering in Medicine and Biology Society*, pages 3676–3679. IEEE, 2012.

Michael Wibral, Nicolae Pampu, Viola Priesemann, Felix Siebenhühner, Hannes Seiwert, Michael Lindner, Joseph T Lizier, and Raul Vicente. Measuring information-transfer delays. *PloS one*, 8(2):e55809, 2013.

Michael Wibral, Raul Vicente, and Joseph T Lizier. *Directed information measures in neuroscience*. Springer, 2014.

Patricia Wollstadt, Joseph T Lizier, Raul Vicente, Conor Finn, Mario Martinez-Zarzuela, Pedro Mediano, Leonardo Novelli, and Michael Wibral. IDTxl: The Information Dynamics Toolkit xl: a Python package for the efficient analysis of multivariate information dynamics in networks. *arXiv preprint arXiv:1807.10459*, 2018.
